# Nanofractionation Analytics for Comparing MALDI-MS and ESI-MS Data of *Viperidae* Snake Venom Toxins

**DOI:** 10.3390/toxins16080370

**Published:** 2024-08-21

**Authors:** Haifeng Xu, Jesse Mastenbroek, Natascha T. B. Krikke, Susan El-Asal, Rama Mutlaq, Nicholas R. Casewell, Julien Slagboom, Jeroen Kool

**Affiliations:** 1 Department of Chemistry and Pharmaceutical Sciences, Division of BioAnalytical Chemistry, Faculty of Science, Amsterdam Institute of Molecular and Life Sciences, Vrije Universiteit Amsterdam, De Boelelaan 1085, 1081 HV Amsterdam, The Netherlands; 2 Centre for Analytical Sciences Amsterdam (CASA), 1012 WX Amsterdam, The Netherlands; 3 Centre for Snakebite Research & Interventions, Liverpool School of Tropical Medicine, Liverpool L3 5QA, UK

**Keywords:** viperid venoms, ESI-MS, MALDI-MS, intact MS comparison, plasma coagulation

## Abstract

Worldwide, it is estimated that there are 1.8 to 2.7 million cases of envenoming caused by snakebites. Snake venom is a complex mixture of protein toxins, lipids, small molecules, and salts, with the proteins typically responsible for causing pathology in snakebite victims. For their chemical characterization and identification, analytical methods are required. Reversed-phase liquid chromatography coupled with electrospray ionization mass spectrometry (RP-LC-ESI-MS) is a widely used technique due to its ease of use, sensitivity, and ability to be directly coupled after LC separation. This method allows for the efficient separation of complex mixtures and sensitive detection of analytes. On the other hand, matrix-assisted laser desorption/ionization mass spectrometry (MALDI-MS) is also sometimes used, and though it typically requires additional sample preparation steps, it offers desirable suitability for the analysis of larger biomolecules. In this study, seven medically important viperid snake venoms were separated into their respective venom toxins and measured by ESI-MS. In parallel, using nanofractionation analytics, post-column high-resolution fractionation was used to collect the eluting toxins for further processing for MALDI-MS analysis. Our comparative results showed that the deconvoluted snake venom toxin masses were observed with good sensitivity from both ESI-MS and MALDI-MS approaches and presented overlap in the toxin masses recovered (between 25% and 57%, depending on the venom analyzed). The mass range of the toxins detected in high abundance was between 4 and 28 kDa. In total, 39 masses were found in both the ESI-MS and/or MALDI-MS analyses, with most being between 5 and 9 kDa (46%), 13 and 15 kDa (38%), and 24 and 28 kDa (13%) in size. Next to the post-column MS analyses, additional coagulation bioassaying was performed to demonstrate the parallel post-column assessment of venom activity in the workflow. Most nanofractionated venoms exhibited anticoagulant activity, with three venoms additionally exhibiting toxins with clear procoagulant activity (*Bothrops asper*, *Crotalus atrox*, and *Daboia russelii*) observed post-column. The results of this study highlight the complementarity of ESI-MS and MALDI-MS approaches for characterizing snake venom toxins and provide a complementary overview of defined toxin masses found in a diversity of viper snake venoms.

## 1. Introduction

Venom is a chemical weapon used by many species across the animal kingdom, with some of the venoms most medically relevant to humans found in snakes. While venomous snakes primarily use venom for prey capture, it can be deployed defensively, and it is in this context that human snakebite occurs. Every year, approximately 5.4 million people suffer from snakebite, resulting in up to 2.7 million cases of envenoming. These envenomings can cause severe long-term health implications, such as amputations or other permanent disabilities, in perhaps as many as 400,000 people a year [1]. However, it is thought that an additional 138,000 victims suffer mortality as the result of the bite, and the greatest burden falls on those based in the tropics. Because of this, the World Health Organization added snakebite to their priority list of neglected tropical diseases in 2017 [2,3,4].

Snakebite pathology is the result of the hemotoxic, cytotoxic, or neurotoxic properties of snake venom toxins [5], which may cause local or systemic damage, including necrosis, hemorrhage, and respiratory paralysis [3]. Many types of compounds are present in snake venom, including inorganic cations, lipids and carbohydrates. The toxic properties, however, are mainly caused by proteins and peptides, which are highly abundant in snake venom (>90% dry weight) [6]. These toxins vary extensively between snakes, of which the most prevalent and medically significant are members of the families *Elapidae* (elapids) and *Viperidae* (vipers), the latter of which is divided into the *Viperinae* (true vipers) and *Crotalinae* (pit vipers) subfamilies. Within the vipers, the dominant protein toxin families are phospholipases A_2_ (PLA_2_s), snake venom metalloproteinases (SVMPs), and snake venom serine proteases (SVSPs). Secondary toxin families include Kunitz peptides, L-amino acid oxidases, cysteine-rich secretory proteins, C-type lectins, disintegrins, and natriuretic peptides [7,8].

The toxin bioactivities of viper venoms have been thoroughly identified for many decades, both at the level of crude venom and purified toxins. From these studies, we know that SVSPs can affect hemostasis through their influence on platelet aggregation, blood coagulation, and fibrinolysis [9,10]. In contrast, SVMPs can cause local and systemic hemorrhage via the cleavage of basement membrane proteins and coagulopathy via activation of key clotting factors (prothrombin, factor X) [11]. These proteases are also associated with other pharmacological actions, such as myotoxicity and inflammation [12,13]. Additionally, synovial-type group II PLA_2_s are prevalent in most vipers [14]. These lipid-hydrophilizing enzymes display a variety of pharmacological functions: acidic PLA_2_s inhibit platelet aggregation, whereas basic PLA_2_s are myotoxic, neurotoxic, and/or anticoagulant in nature [15].

Advances in snake venom analysis have revolutionized our understanding of the complex compositions and biological activities of venoms [16]. One significant area of progress is in venom separation techniques, where high-resolution analytical chromatographic methods such as reversed-phase liquid chromatography (RPLC), size-exclusion chromatography (SEC), and cation exchange chromatography have been used to fractionate venom components based on their physicochemical properties [17,18,19,20]. Subsequently, mass spectrometry (MS) analysis is often directly (i.e., online or hyphenated) or indirectly (i.e., offline) conducted, enabling the measurement of accurate masses of individual venom proteins and peptides [21,22,23]. In parallel, post-column bioassay screening approaches have been combined by, for example, nanofractionation analysis for post-column offline screening using biochemical assays to elucidate the cytotoxicity, neurotoxicity, and hemotoxicity of venom toxins [21,24,25]. Other examples include venom toxin profiling for its effects on ion channels, enzymes, and cellular signaling pathways [26,27,28]. And interaction with antivenom treatment, i.e., antivenomics [29]. LC coupled online to MS has been described for several years [30], and MS analysis after electrospray ionization (ESI) following LC separation is the most well-known approach.

ESI is a widely used technique in MS for the analysis of complex biological samples, including snake venoms. ESI allows for the efficient ionization of venom toxins, enabling their detection and characterization [31,32,33]. While LC-ESI-MS is most often used for intact toxin analysis of toxins in venoms, matrix-assisted laser desorption/ionization MS (MALDI-MS), which is also a soft ionization technique, can also be used. However, unlike LC-ESI-MS, MALDI-MS cannot be directly coupled post-column to LC, as after post-separation and fraction collection, off-line sample preparation is required prior to MALDI-MS analysis. In MALDI-MS, the analytes of interest, in this case fractionated snake venom toxins, are mixed with a matrix compound solution and then deposited on a target MALDI plate. After analyte and matrix spotting on the MALDI plate, followed by the drying of the solvents, the MALDI plate is inserted into the mass spectrometer for analysis. The matrix consists of a small organic molecule that absorbs the laser energy of the MALDI source and facilitates the desorption and ionization of the analyte molecules upon laser irradiation enabling efficient ionization of toxins. MALDI-MS is, therefore, much more labor-intensive than LC-ESI-MS and cannot easily be fully automated. Nevertheless, instead of depending solely on one MS method for toxin identification, researchers are increasingly adopting multi-analytical approaches by combining various techniques side by side to obtain more mass spectral information from samples of interest.

In this study, we used LC coupled to nanofractionation analysis for crude venom separation, followed by high-resolution fractionation towards MALDI-MS analysis. In parallel, via a post-column split and prior to fractionation, ten percent of the eluent was directed to online ESI-MS. In addition to performing MALDI-MS on the collected fractions, they were processed for coagulation bioassaying. The analytical setup employed here focused on viper venoms, for which a comparison of measured LC-MALDI-MS and LC-ESI-MS data was made, and integrated with an additional coagulation bioassay. We show that this approach can greatly facilitate the discovery and MS-based identification of bioactive toxins from viper snake venoms.

## 2. Results and Discussion

**Figure 1:**
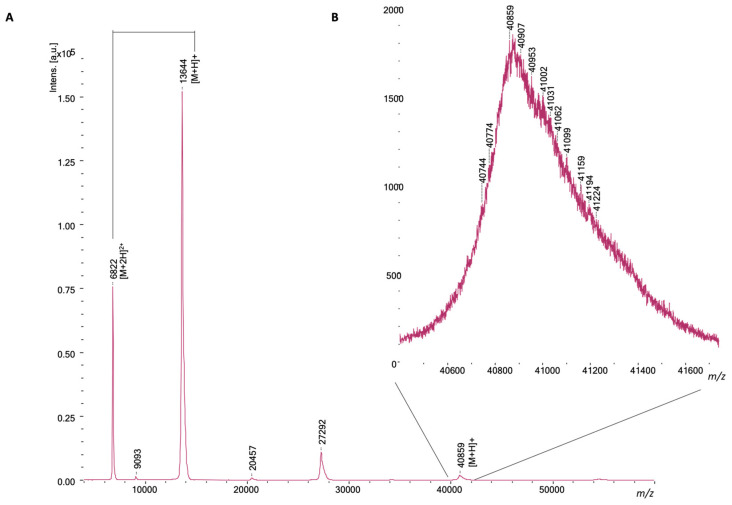
MALDI-MS spectrum obtained for a nanofractionated toxin fraction. (**A**) shows a typical MALDI-MS spectrum obtained for nanofractionated toxins of *D. russelii* venom at the retention time fraction of 20.0 min. The toxin ions with the mass of 13,644 Da presented themselves as singly (M + H)^+^ and doubly charged (M + 2H)^2+^ ions (which were used for toxin mass deconvolution) with *m*/*z* values of 13,644 and 6822 based on manual deconvolution. (**B**) shows zoomed-in toxin-ion peaks with a *m*/*z* of 40,859.

This study investigated the combined use of LC-ESI-MS and LC-MALDI-MS to identify similarities and complementarities in data acquired for viper venom research. Parallel acquired and integrated coagulation bioassaying results were included to demonstrate simultaneous bioassaying capability, and the corresponding MS data collected facilitated the identification of certain coagulopathic toxins. To acquire clear comparisons between the two ionization approaches, Extracted Ion Currents (EICs) were extracted from the ESI-MS data, while the signal intensity of each significant mass in each mass spectrum measured from each MALDI-MS-processed well was plotted on a y-axis versus the retention time of fractionation on the x-axis to create so-called MALDI-MS chromatograms for each toxin. Figure 1A shows a typical MALDI-MS spectrum obtained for nanofractionated toxins from *D. russelii* venom at the retention time fraction of 20.0 min. The toxin ions detected presented as singly (M + H)^+^ and doubly charged (M + 2H)^2+^ ions (which were used for toxin mass deconvolution). Figure 1B shows zoomed-in toxin-ion peaks of the toxin with *m*/*z* of 6822 and 13,644, which were the doubly and singly charged ions, respectively, of the toxin, with a mass of 13,644 Da, based on manual deconvolution.

Throughout this paper, toxin masses detected from both the LC-ESI-MS and LC-MALDI-MS results generated in this study are named “matched” results. Matched results were defined as having mass differences of ±10 Da. Masses only found in the LC-ESI-MS data or the LC-MALDI-MS data are referred to as “unmatched” masses. The “matched” and “unmatched” mass ions of the nanofractionated toxins will be discussed, and correlations will be made with the measured coagulation activity bioassay data to highlight parallel biochemical profiling within the workflow. In total, seven viper venoms were analyzed, and the results for each were addressed in the order of *Bitis arietans*, *Bothrops jararaca*, *Bothrops asper*, *Crotalus atrox*, *Daboia russelii*, *Echis carinatus*, and *Echis ocellatus.*

**Figure 2:**
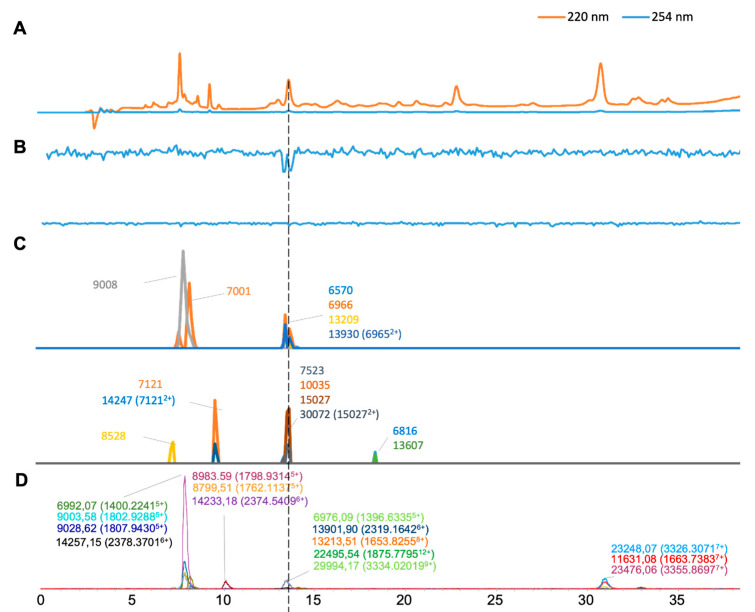
Integrated *Bitis arietans* venom results: superimposed results of LC-UV data, LC-(ESI)-MS data, and nanofractionation MALDI-MS data plotted chromatographically and nanofractionation coagulation bioassay data plotted chromatographically. (**A**) LC-UV trace of separated snake venom at 220 nm (orange) and 254 nm (blue). (**B**) Coagulation bioactivity chromatograms representing anticoagulation (upper trace) and procoagulation activity (lower trace). (**C**) Chromatographically plotted MALDI-MS data of the identified toxins in the wells with nanofractionated toxins. For each toxin identified in different wells, the measured intensity from the MALDI data was plotted on the y-axis versus the retention time of fractionation on the *x*-axis. As all toxins were eluted over a series of subsequent wells, so-called MALDI-MS chromatograms of each toxin were the result. (**D**) Extracted Ion Currents (EICs) from the LC-ESI-MS data.

The results of the *B. arietans* venom analysis are shown in Figure 2, and we found 16 distinct masses from the ESI-MS measurement. For each charge state envelope representing a toxin’s accurate mass, the mass-to-charge (*m*/*z*) value belonging to the highest abundant charge state was selected for plotting the respective toxin’s EIC. Most toxin masses included for comparison were around 6–29 kDa. For the so-called MALDI-MS chromatograms (Figure 2C), we plotted 14 clearly observed masses retrieved from the fractionated toxins (the masses calculated from ion peaks were generated by the Flexanalysis software 3.4). Among those, four masses were matched with the masses of the ESI-MS results, as defined as having mass differences lower than ±10 Da (Table 1). Three doubly charged *m*/*z* (6965, 7121, and 15,027 Da) values were deconvoluted with the accurate masses of 13,930, 14,247, and 30,072 Da. For the bioassay results, only one negative peak representing anticoagulant activity was observed at a retention time of around 13.5 min. The accurate masses corresponding to this biological activity were deconvoluted from both MS methods as 6976.09 Da (6966 Da in MALDI) and 13,213.51 Da (13,209 Da in MALDI). The latter of these masses, which is in the range known for PLA_2_ toxins, was likely responsible for the bioactivity observed.

**Figure 3:**
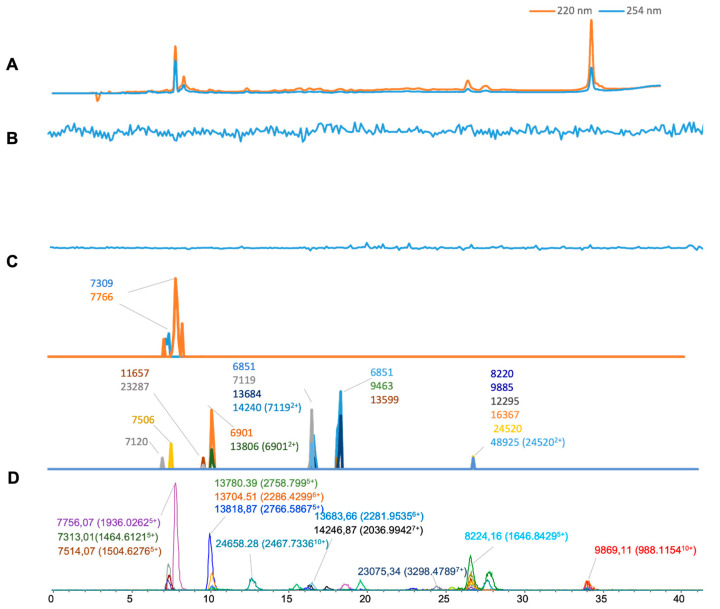
Integrated *Bothrops jararaca* venom results: superimposed results of LC-UV data, LC-(ESI)-MS data, and nanofractionation MALDI-MS data plotted chromatographically and nanofractionation coagulation bioassay data plotted chromatographically. (**A**) LC-UV trace of separated snake venom at 220 nm (orange) and 254 nm (blue). (**B**) Coagulation bioactivity chromatograms representing anticoagulation (upper trace) and procoagulation activity (lower trace). (**C**) Chromatographically plotted MALDI-MS data of the identified toxins in the wells with nanofractionated toxins. For each toxin identified in different wells, the measured intensity from the MALDI data was plotted on the y-axis versus the retention time of fractionation on the *x*-axis. As all toxins were eluted over a series of subsequent wells, so-called MALDI-MS chromatograms of each toxin were the result. (**D**) Extracted Ion Currents (EICs) from the LC-ESI-MS data.

Figure 3 shows the results of the analysis of *B. jararaca* venom. In Figure 3D, 22 distinct masses found from the ESI-MS measurement of this venom are presented with the highest intensity *m*/*z* value of the related charge states plotted. Most toxin masses included for comparison were around 6–27 kDa. For the so-called MALDI-MS chromatograms (Figure 3C), we plotted 20 clearly observed masses. Among those, six masses were matched with masses from the ESI-MS (Table 1). Three doubly charged *m*/*z* values (6901, 7119, and 24,520) were deconvoluted to the accurate masses of 13,806, 14,240, and 48,925 Da, respectively. For the bioassay results, no significant anticoagulant or procoagulant peaks were observed. Based on previous studies on the same venom [34], it is likely that the procoagulant proteases present in this venom denatured during the chromatographic separation or that the venom concentration used was insufficient to yield a strong signal in the bioassay.

**Figure 4:**
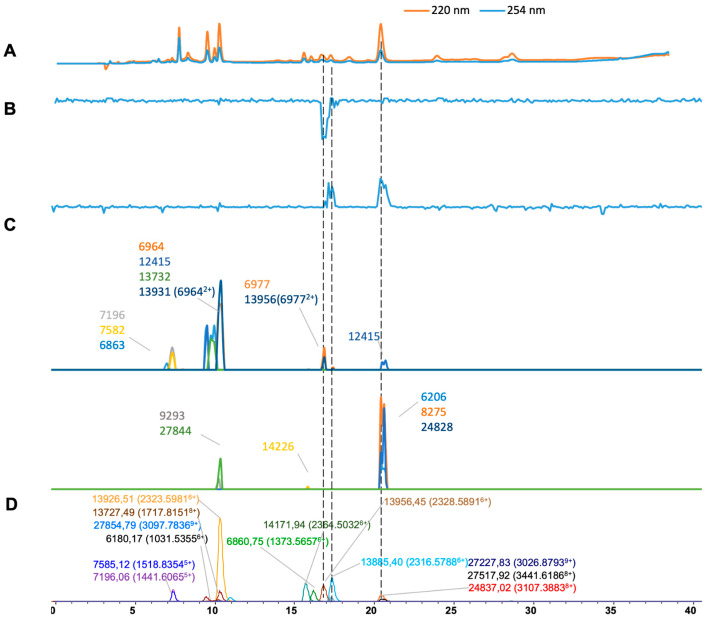
Integrated *Bothrops asper* venom results: superimposed results of LC-UV data, LC-(ESI)-MS data, and nanofractionation MALDI-MS data plotted chromatographically and nanofractionation coagulation bioassay data plotted chromatographically. (**A**) LC-UV trace of separated snake venom at 220 nm (orange) and 254 nm (blue). (**B**) Coagulation bioactivity chromatograms representing anticoagulation (upper trace) and procoagulation activity (lower trace). (**C**) Chromatographically plotted MALDI-MS data of the identified toxins in the wells with nanofractionated toxins. For each toxin identified in different wells, the measured intensity from the MALDI data was plotted on the y-axis versus the retention time of fractionation on the *x*-axis. As all toxins were eluted over a series of subsequent wells, so-called MALDI-MS chromatograms of each toxin were the result. (**D**) Extracted Ion Currents (EICs) from the LC-ESI-MS data.

For *Bothrops asper* (Figure 4), we extracted 13 distinct masses from ESI-MS (Figure 4D), and14 masses were deconvoluted from MALDI-MS and plotted (Figure 4C). Of these, eight masses were matched from the two MS methods (Table 1). Two doubly charged *m*/*z*-values (6977 and 6964) were deconvoluted to the masses of 13,956 and 13,931 Da, respectively. As for the results of the plasma coagulation assay, procoagulant activity was observed as positive peaks at retention times of 17.5 and 20.5 min, corresponding to the masses of 13,885.40 and 24,837.02 Da in ESI-MS, respectively (only 24,828 Da eluted at 20.5 min was found for the MALDI-MS results). The significant anticoagulant peak observed at a retention time of 17.0 min correlated with the mass of 13,956.45 Da (13,956 in MALDI).

**Figure 5:**
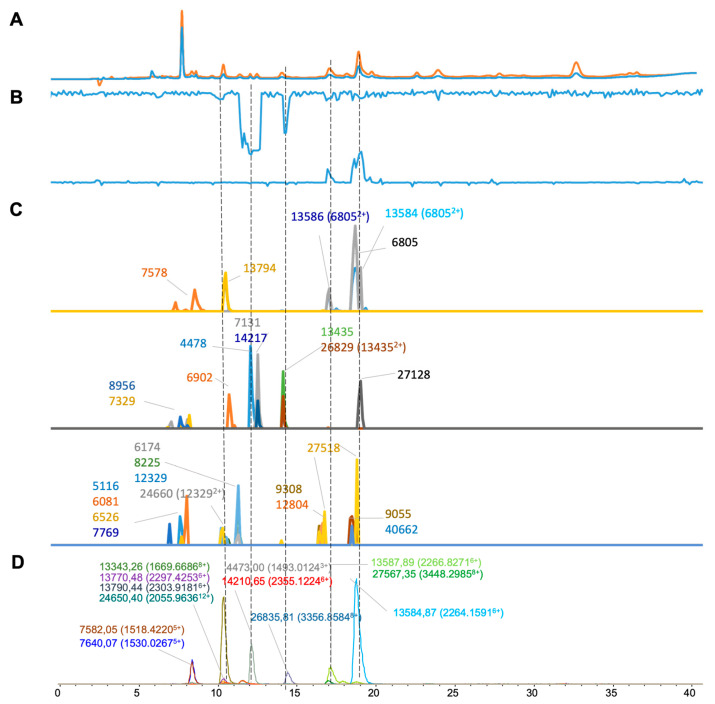
Integrated *Crotalus atrox* venom results: superimposed results of LC-UV data, LC-(ESI)-MS data, and nanofractionation MALDI-MS data plotted chromatographically and nanofractionation coagulation bioassay data plotted chromatographically. (**A**) LC-UV trace of separated snake venom at 220 nm (orange) and 254 nm (blue). (**B**) Coagulation bioactivity chromatograms representing anticoagulation (upper trace) and procoagulation activity (lower trace). (**C**) Chromatographically plotted MALDI-MS data of the identified toxins in the wells with nanofractionated toxins. For each toxin identified in different wells, the measured intensity from the MALDI data was plotted on the y-axis versus the retention time of fractionation on the *x*-axis. As all toxins were eluted over a series of subsequent wells, so-called MALDI-MS chromatograms of each toxin were the result. (**D**) Extracted Ion Currents (EICs) from the LC-ESI-MS data.

For the results of *Crotalus atrox* venom (Figure 5), 12 distinct masses were deconvoluted from ESI-MS (Figure 5D) and 22 masses were deconvoluted from MALDI-MS (Figure 5C). From there, six masses were found to be shared across both MS methods (Table 1). Two doubly charged *m*/*z* values (13,435 and 12,329) were deconvoluted to masses of 26,829 and 24,660 Da, respectively. For bioassaying, both procoagulant and anticoagulant activity were observed for the nanofractionated *C. atrox* venom. Two notable negative peaks were observed showing anticoagulant effects at retention times of 12.0 and 14.5 min and had corresponding ESI-MS masses of 14,210.65 Da (14,217 Da in MALDI-MS) and 26,835.81 Da (26,829 Da in MALDI-MS), respectively. Procoagulant bioactivity peaks were observed at retention times of 17.0 and 19.0 min, which corresponded to the ESI-MS-derived accurate masses of 13,587.89 Da (13,586 Da in MALDI-MS) and 13,584.87 Da (13,584 in MALDI-MS), respectively.

**Figure 6:**
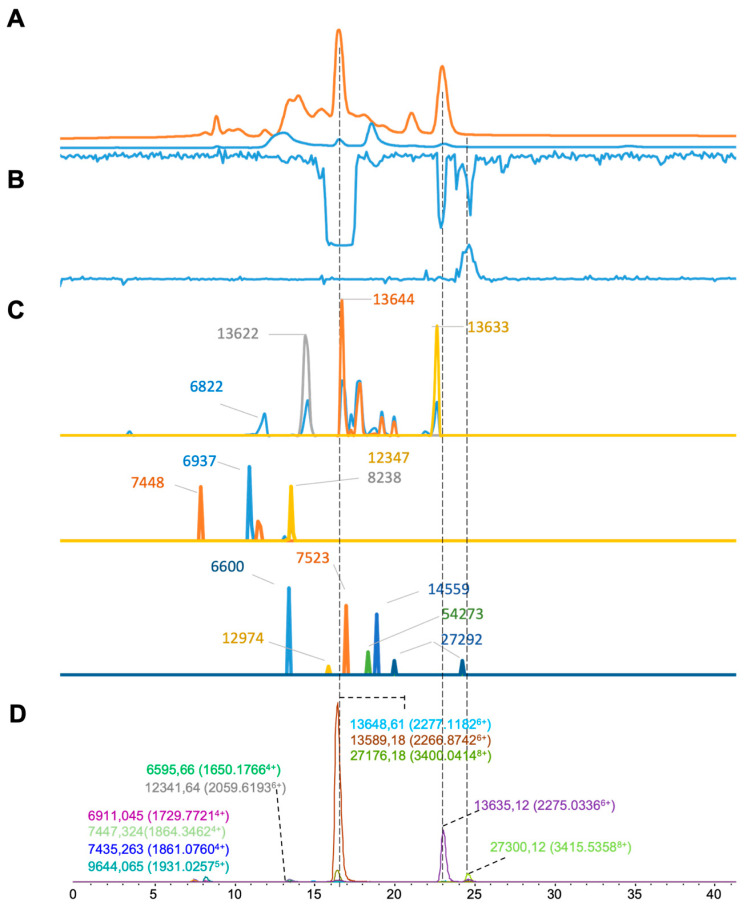
Integrated *Daboia russelii* venom results: superimposed results of LC-UV data, LC-(ESI)-MS data, and nanofractionation MALDI-MS data plotted chromatographically and nanofractionation coagulation bioassay data plotted chromatographically. (**A**) LC-UV trace of separated snake venom at 220 nm (orange) and 254 nm (blue). (**B**) Coagulation bioactivity chromatograms representing anticoagulation (upper trace) and procoagulation activity (lower trace). (**C**) Chromatographically plotted MALDI-MS data of the identified toxins in the wells with nanofractionated toxins. For each toxin identified in different wells, the measured intensity from the MALDI data was plotted on the y-axis versus the retention time of fractionation on the *x*-axis. As all toxins were eluted over a series of subsequent wells, so-called MALDI-MS chromatograms of each toxin were the result. (**D**) Extracted Ion Currents (EICs) from the LC-ESI-MS data.

For the results of *Daboia russelii* venom (Figure 6), 11 distinct masses were deconvoluted from the ESI-MS data (Figure 6D), and 15 were deconvoluted from MALDI-MS (Figure 6C). Of these, six masses that matched were found by both MS methods (Table 1). As for the plasma coagulation assay results, both procoagulant and anticoagulant activity were observed (Figure 6B). Three notable negative anticoagulant peaks were found at retention times of 16.5, 23.0 and 25.0 min, where their corresponding masses by ESI-MS were 13,648.61 Da (13,644 Da in MALDI-MS), 13,635.12 Da (13,633 Da in MALDI-MS), and 27,300.12 Da (27,292 Da in MALDI), respectively. In addition, a procoagulant venom peak was observed at a retention time of 24.0–26.0 min, which corresponded to the accurate masses of 27,300.12 Da determined by ESI-MS and 27,292 Da determined by MALDI-MS.

**Figure 7:**
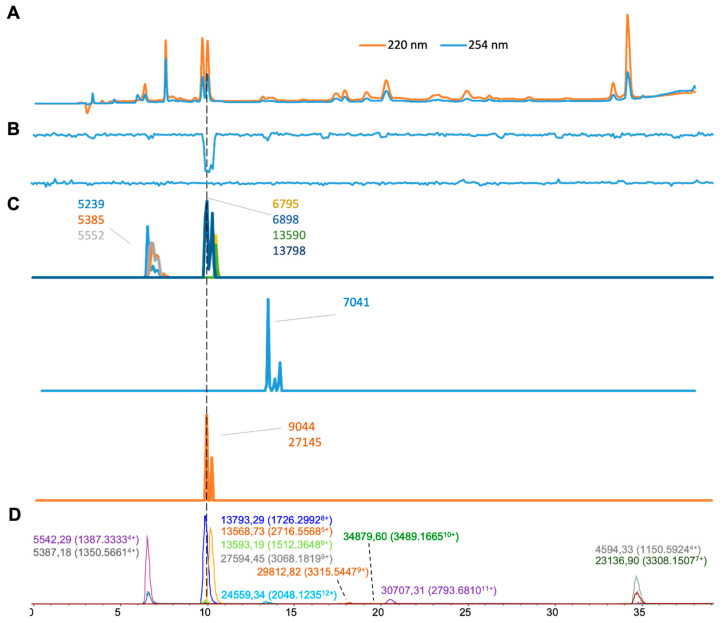
Integrated *Echis carinatus* venom results: superimposed results of LC-UV, LC-(ESI)-MS, and nanofractionated MALDI-MS data plotted chromatographically and nanofractionated coagulation bioassay data plotted chromatographically. (**A**) LC-UV trace of separated snake venom at 220 nm (orange) and 254 nm (blue). (**B**) Coagulation bioactivity chromatograms representing anticoagulation (upper trace) and procoagulation activity (lower trace). (**C**) Chromatographically plotted MALDI-MS data of the identified toxins in the wells with nanofractionated toxins. For each toxin identified in different wells, the measured intensity from the MALDI data was plotted on the y-axis versus the retention time of fractionation on the *x*-axis. As all toxins were eluted over a series of subsequent wells, so-called MALDI-MS chromatograms of each toxin were the result. (**D**) Extracted Ion Currents (EICs) from the LC-ESI-MS data.

For *Echis carinatus* venom (Figure 7), we deconvoluted 13 and 10 distinct, clearly observed masses from the results of the ESI-MS and MALDI-MS experiments, respectively (Figure 7C,D). Among those, five masses were matched across both approaches (Table 1). Only one anticoagulant peak was observed (Figure 7B) at a retention time of 10.0 min, which corresponded to two accurate masses of 13,793.29 Da (13,798 Da in MALDI-MS) and 13,593.18 Da (13,590 Da in MALDI). These toxins seem mostly likely to be PLA_2_ toxins, given the detected mass. While no procoagulant venom activity was detected in the bioassay, it is likely that the procoagulant proteases present in this venom denatured during chromatographic separation.

**Figure 8:**
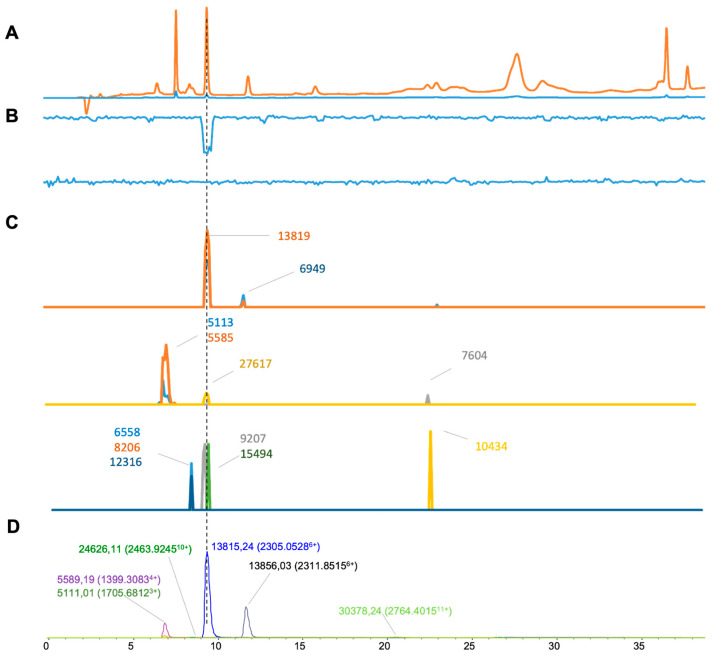
Integrated *Echis ocellatus* venom results: superimposed results of LC-UV, LC-(ESI)-MS, and nanofractionated MALDI-MS data plotted chromatographically and nanofractionated coagulation bioassay data plotted chromatographically. (**A**) LC-UV trace of separated snake venom at 220 nm (orange) and 254 nm (blue). (**B**) Coagulation bioactivity chromatograms representing anticoagulation (upper trace) and procoagulation activity (lower trace). (**C**) Chromatographically plotted MALDI-MS data of the identified toxins in the wells with nanofractionated toxins. For each toxin identified in different wells, the measured intensity from the MALDI data was plotted on the y-axis versus the retention time of fractionation on the *x*-axis. As all toxins were eluted over a series of subsequent wells, so-called MALDI-MS chromatograms of each toxin were the result. (**D**) Extracted Ion Currents (EICs) from the LC-ESI-MS data.

The final venom tested was that of *Echis ocellatus* (Figure 8), for which we extracted six distinct masses from ESI-MS (Figure 8D), and ten masses were deconvoluted from MALDI-MS (Figure 8C). From there, three masses were matched within both MS methods (Table 1). As for the plasma coagulation assay results (Figure 8B), anticoagulant activity was seen as a negative peak at a retention time of 9.3 min corresponding to a mass of 13,815.24 Da in ESI-MS (13,819 Da in MALDI-MS), which again likely represents a PLA_2_ toxin. As with *E. carinatus*, no procoagulant venom activity was observed, which, given the nature of this procoagulant venom, is almost certainly due to proteases denaturing during the chromatographic separation step.

The venom of viper snakes represents complex mixtures of toxins that exhibit diverse structures and bioactivities. With a better understanding of the toxins in venoms, their variability, and envenoming mechanisms, new pathology- and/or target-specific snakebite treatments may be developed [35]. Moreover, it may give insights into snake evolution [36] and might even result in the discovery of new (bio)pharmaceuticals [37,38].

## 3. Discussion

In this study, we presented a comparison of analytical results by investigating data obtained from several viper venoms analyzed by both ESI-MS and MALDI-MS approaches. In addition, post-column plasma coagulation screening was included in this study to assess bioassay integration. This study involved results obtained from seven geographically diverse and medically important viper species, specifically *B. arietans*, *B. jararaca*, *B. asper*, *C. atrox*, *D. russelii*, *E. carinatus*, and *E. ocellatus*. For the analysis using MALDI-MS, we permitted a 10 Da cut-off limit to determine whether deconvoluted masses matched with corresponding accurate masses retrieved from the ESI-MS data. Similar mass errors for MALDI-TOF-MS, as used in our study, were also used in previous studies (shown in Table 2). In general, the deconvoluted masses from both ESI-MS and MALDI-MS presented an overlap in masses found, ranging from between 25% and 57% of toxins detected, depending on the venom analyzed (Table 1). When looking at the matching masses found for both methods, the focus was on clearly observed ions in the MS spectra. In contrast to ESI-MS, MALDI-MS required labor-intensive manual effort during both the sample preparation and data processing phases. In total, 39 good-intensity-observed matching masses were found across the resulting datasets. Most matching masses were found to be around 5–9 kDa (28.2%; calculated by the number of deconvoluted masses from 5 to 9 kDa in MALDI-MS/the total matched masses in MALDI-MS × 100%) and 13–15 kDa (38.4%) kDa. Five good-intensity toxins were found with masses around 24–28 kDa. These masses were 24,837.02 Da (24,828 Da in MALDI-MS) and 27,854.79 Da (27,844 Da in MALDI-MS) for *B. asper* venom, 26,835.81 Da (26,829 Da in MALDI-MS) and 24,650.4 Da (24,660 in MALDI-MS) for *C. atrox* venom, and 27,300.12 Da (27,292 Da in MALDI-MS) for *D. russelii* venom. While considering the complex composition and significant venom variations in *Viperidae* species, for certain mass ranges, it is difficult to define the toxin family only by its molecular mass. For full identification of all toxins in each venom analyzed, additional proteomics studies have to be conducted. These studies represent standalone elaborate studies and were out of the scope for the current study in which a new analytical approach for venom research was presented. For the venoms included in our study, venom proteomics studies have been performed and published. These proteomics studies comprehensively analyzed one or several venoms with, as a core focus, the elucidation of the venom’s toxin composition. For instance, Wagstaff et al. characterized the venom proteome and the venom gland transcriptome of *Echis ocellatus* to provide a detailed compositional analysis for designing novel toxin-specific immunotherapies [39]. Alape-Girón et al. performed a comparative proteomic characterization of the geographic variation in *Bothrops asper* venom from Central America and northern South America [40]. Calvete et al. used venomics approaches to gain an insight into the overall *Crotalus atrox* venom proteome [41]. Sharma et al. unveiled the complexities of *Daboia russelii* venom in their venomics study and identified 63 different proteins belonging to 12 families [42]. Dingwoke et al. analyzed the venom of *Bitis arietans* by a venomics method, which resulted in the identification of 79 toxins belonging to 14 families [43]. Zelanis et al. studied the sex-dependent variation in the *Bothrops jararaca* proteome and highlighted the role of glycosylation as a key PTM (post-translational modification) contributing to variability through a combination of SDS-PAGE and 2-DE and through shotgun proteomic analysis [44]. Patra et al. carried out a venomics study to analyze the *Echis carinatus* proteome and found large differences when using different databases for processing their proteomics MS data [45]. Our focus was on developing a preliminary methodology for the chromatographic comparisons and alignments of several analytical datasets for screening Viperid venoms, which can broaden the analytical toolbox for toxicovenomics research. We did not perform any MS/MS (MS2) experiments or bottom-up proteomics experiments and only acquired toxin masses next to toxin bioactivities. Adding proteomics data to our study would deviate from our focus and would make this study way too lengthy. As such, it is not possible to correlate our data with previously published proteomics studies. Nevertheless, including proteomics data in future studies would be a valuable advancement of the here-presented research, in which the proteomics data allows for actual (bioactive) toxin identification, i.e., bottom-up, top-down, Edman degradation, or a combination of these [46]. As for the coagulation bioactivity data, the venoms of most species, except *B. jararaca*, were found to have procoagulant and/or anticoagulant activity. While anticoagulant effects were observed in all six remaining venoms, clear procoagulant effects were only observed in the nanofractionated venoms of *B. asper*, *C. atrox*, and *D. russelii.* Given that metalloproteinase and serine protease enzymes have been demonstrated to have thrombin-like activity, platelet aggregation activity, and coagulation factor activating activities and are typically found in viper venoms [10,13,47], it was anticipated that procoagulant toxins would be observed in all the samples tested. However, for the venoms of *E. carinatus* and *E. ocellatus*, only anticoagulant effects were seen at our tested venom concentration of 1 mg/mL. Our previous studies investigating the bioactivity of these two venoms, such as the study by Xie et al. [34], previously detected procoagulant effects, though higher venom concentrations of up to 5 mg/mL were used. It seems likely that the lower venom concentrations applied here, coupled with these larger proteins at least partially denaturing during venom separation by RP-LC, are responsible for the disparities in our results.

The presented analytical approach has a number of current limitations. The methodology gives non-quantitative toxin data. The MS data are only valuable for assessing toxin accurate masses and cannot be used for quantitative purposes since toxin sensitivity is different for each toxin and is highly dependent on many factors, among others a toxin’s ionization efficiency (which, in turn, is dependent on its structure). Based on LC-UV chromatogram peak areas, we, however, can make a crude overview of relative toxin abundances at the level of low, medium, or high abundance in the venom analyzed. The analytical methodology was narrowed down to a limited number of toxin masses to be correlated to the bioactivity peaks observed. However, for chromatographic separation, insufficient separation of toxins in several cases resulted in close co-elution, thereby making it hard to pinpoint what exact toxin caused a bioactivity. Also, toxin denaturation can happen during separation. For the ESI-MS data, when using a standard ESI-Q-TOF-MS coupled to RP-HPLC separations, charge-state distributions change to higher numbers upon increasing toxin masses. Among other effects, this results in sensitivity decreases due to toxin mass increases. For the analysis of MALDI-MS, limitations include the fact that mass resolution was limited to 10 Da, and in cases where no two charge stages were found for a toxin, it was difficult to deconvolute the *m*/*z* values found from the MS data. Producing the plotted MALDI-MS chromatograms is a manual, time-consuming, and laborious task. Future progress in this regard can include scripts to make it easier to process this data at a higher throughput. Although the focus was on the toxins detected with good signal abundance, not many higher-mass-range toxins were found to have a good signal abundance. Some were found in the MALDI-MS data, but with very low abundance. The Supplementary Materials PowerPoint document “Superimposed MALDI-MS spectra of all toxin fractions analyzed for each venom.pptx” gives the MALDI-MS spectral data of all the fractionated toxins analyzed.

## 4. Conclusions

In this study, we provided a chromatographic comparison of analytical results by investigating data obtained from several viper venoms analyzed by both ESI-MS and MALDI-MS approaches. In addition, post-column plasma coagulation screening was included in this study to assess bioassay integration. To finalize, the present work gave chromatographic analytical alignments that showed promising toxin identification results for viper venom analysis by combining both MS methods, which enriched the analytical toolbox for snake toxicovenomics research.

## 5. Experimental

### 5.1. Reagents

Calcium chloride (CaCl_2_) was purchased from Sigma-Aldrich (Zwijndrecht, The Netherlands). Bovine plasma (500 mL, Sodium Citrated, Sterile Filtered, Product Code: S0260) was purchased from Biowest (Nuaillé, France). α-cyano-4-hydroxycinnamic acid was purchased from Sigma Aldrich (Zwijndrecht, The Netherlands). Acetonitrile (ACN) and trifluoroacetic acid (TFA, MS grade) were purchased from Biosolve (Valkenswaard, The Netherlands) and were of UPLC/MS grade. Water used in this study was sourced from a Milli-Q plus system (Millipore, Amsterdam, The Netherlands).

### 5.2. Venoms

The venoms of *Bitis arietans* (captive bred, Nigeria), *Bothrops jararaca* (captive bred, Brazil), *Bothrops asper* (Central American lancehead, Costa Rica), *Crotalus atrox* (Western diamondback rattlesnake, USA), *Daboia russelii* (Russell’s viper, Sri Lanka), *Echis carinatus* (Indian saw-scaled viper, India), and *Echis ocellatus* (West African carpet viper, Nigeria) were stored as freeze-dried powders at 4 °C until use. These lyophilized venoms were provided by the Centre for Snakebite Research and Interventions at the Liverpool School of Tropical Medicine, UK, either from wild-caught snakes maintained in the herpetarium or from the historical venom stocks held in this facility. Crude venom solutions (1 ± 0.1 mg/mL) were prepared in MQ water as stock solution aliquots and then stored at −80 °C until use.

### 5.3. Nanofractionation Analytics

Liquid chromatography separation, nanofractionation, and parallel MS analysis were performed by a Shimadzu HPLC system. A Shimadzu SIL-20AC autosampler was set at a 50 μL injection volume. The Shimadzu LC-20AB pump was set at a total flow rate of 0.5 mL/min. The Shimadzu SPD-20A UV detector was set at 220 nm for UV detection. The samples were separated on a Waters XBridge Peptide BEH C18 column (4.6 × 100 mm; 5 μm; 300 Å) with an XBridge Peptide BEH C18 Guard Column (130 Å, 5 µm, 10 mm × 10 mm). The separation was carried out at 30 °C in a Shimadzu CTO-10AC column oven. Mobile phase A was composed of 98% H_2_O, 2% ACN, and 0.1% TFA, while mobile phase B was 98% ACN, 2% H_2_O, and 0.1% TFA. The gradient started at 0% mobile phase B, after which the mobile phase B concentration increased to 13% in the first 5 min and then was linearly increased to 50% within a time period of 30 min, followed by a linear increase from 50% to 90% B within 3 min and then a 7 min isocratic elution at 90% B. Next, mobile phase B was decreased linearly to 0% in 1 min, and the column was then equilibrated for 5 min at 0% B. All settings of the system were controlled by Shimadzu Lab Solutions software 1.0. After separation and UV detection, the elute was split into a 1:9 ratio, of which the 90% fraction was transferred to a FractioMate^TM^ nanofractionator (VU, Amsterdam, The Netherlands) controlled by FractioMator software (V1.0, VU, Amsterdam, The Netherlands). Fractions were collected at a resolution of 6 s/well onto transparent 384-well plates (F-bottom, rounded square well, polystyrene, without lid, clear, non-sterile; Greiner Bio One, Alphen aan den Rijn, The Netherlands). After fragmentation, the well plates were frozen and then vacuum-centrifuged to dryness overnight using an RVC 2-33 CD plus maxi concentrator (Salm en Kipp) with a rotation speed of 1500 rpm, pressure of 1.0 mbar, and temperature of 30 °C. After solvent evaporation, the dried plates were stored at −80 °C prior to further analysis. The smaller fraction was sent to UV detection, which was followed by mass spectrometry detection (or the effluent was sent to the waste after UV detection). Each nanofractionation analytics workflow procedure for analyzing each venom was performed in duplicate.

### 5.4. MALDI-MS Measurements

First, 10 μL MQ was added to the wells of the well plates with fractionated and dried venom toxins. In order to dissolve the venom toxins in MQ, the plates were gently stirred on an automatic stirrer for 10 min at room temperature. Based on the LC-UV data and the retention times of the UV detectable peaks, the wells of interest (i.e., those wells that contained collected fractions that showed UV absorbance upon elution) were selected for MALDI-MS.

For this, 1 μL portions were taken from these wells and deposited on a 384-spot MALDI plate (MTP 384 target ground steel BC, Bruker, Germany). Then, 1 μL of the matrix solution was added to the deposited 1 μL portion fractions, followed by manual aspiration and dispensing for three times for mixing purposes. For the matrix solution, 10 mg/mL of α-Cyano-4-hydroxycinnamic acid (CHCA) was used in a TA50 solution of 50% ACN and 50% MQ and 0.1% TFA. An analysis of the prepared ground steel MALDI plate was performed using an ultrafleXtreme MALDI-TOF/TOF mass spectrometer (Bruker, Germany). Prior to analysis, the MS was calibrated using a peptide mixture containing bradykinin (1–7), angiotensin I, angiotensin II, substance p, bombesin, renin substrate, ACTH 1–17, ACTH 18–39, and somatostatin in a *m*/*z* range from 5000 to 55,000. The MS parameters set were as follows: source 1, 20 kV; source 2, 18.9 kV; lens voltage, 6.5 kV; laser repetition rate, 2 kHz. A total of 15,000 shots for each sample were accumulated, with the laser power gradually increasing from 60 to 100%. FlexControl (Bruker, Germany) was used for instrument control and raw data collection. FlexAnalysis 3.4 (Bruker, Germany) and Microsoft Excel were used to process the data. The ions found in the MALDI-MS spectra were used to construct chromatogram-like graphs by plotting each ion found with significantly high intensity for each 6 s fraction, i.e., its S/N value (on the *y*-axis), versus the fraction’s retention time (on the *x*-axis). As the toxins were all eluted in a subsequent series of adjacent fractions, the chromatographic representations for each toxin found from the MALDI-MS data were the output. These chromatographic representations were compared with the EICs from the ESI-MS analyses. MALDI-MS usually yields singly and doubly positively charged ions. If only one ion was found for a toxin in MALDI-MS, the parallel acquired ESI-MS data were investigated to compare data, and from there the charge state of the ion found in MALDI-MS was assigned.

### 5.5. Online (LC-)ESI-MS Measurements

After the flow split, the 10% split of the LC elute was sent to MS detection (Impact II QTOF, Bruker Daltonics, Germany) with electrospray ionization (ESI) in positive-ion mode at a mass range between 800 and 5000 *m*/*z*. Bruker Compass software 3.4 was used for instrument control and data analysis. For MS data processing, the total-ion current (TIC) was plotted from the recorded MS data. Extracted-ion currents (EICs) were extracted from the TIC by plotting the EIC of the most abundant charge state of each toxin. The ESI source parameters were the capillary voltage of 3.5 kV, source temperature of 200 °C, nebulizer at 0.8 Bar, and dry gas flow of 6.0 L/min. In-source collision-induced dissociation (CID) was set at 200 eV, and 1 average spectrum was stored per second.

After the deconvolution of the masses of the toxins from the MS data, for all the venom toxins analyzed for masses falling within the tentative mass range of PLA_2_ toxins, from Uniprot, the amino acid sequences corresponding to PLA_2_s in the species analyzed were taken from which exact masses were recalculated, taking the cysteine bridges in PLA_2_s into account with the help of Chemdraw. Next, the accurate masses acquired from the MS data could be compared with the calculated candidate exact masses from the Uniprot information.

### 5.6. Delay Time Calculation between LC-UV, LC-MS, and LC-Nano Fractionation

A coagulation bioassay was performed to calculate the delay between the UV data, the MS data, and the fractions collected by the FractioMate. The anticoagulant argatroban was injected, separated by the LC system, and fractionated in a 384-well plate following vacuum centrifugation to dryness. This was followed by a coagulation assay (see the next section below) on the well plate. Where argatroban was eluted, a decreased signal was absorbed in the coagulation assay. By correlating the retention times of the argatroban peak in LC-UV, LC-MS, and the LC-bioassay, the delay times could be determined and then corrected for data analysis. The concentration of argatroban injected was 1 mg/mL with an injection volume of 20 μL. A typical result from the argatroban control analyses is given in the Supplementary Materials “Argatroban (anticoagulant compound)” analyzed by nanofractionation analytics.pptx.

### 5.7. Coagulation Bioassay

For bioassaying, an aliquoted tube with plasma was first thawed in a warm water bath to room temperature. The bioassay protocol was performed based on previous research [23,51,52]. A CaCl_2_ solution (20 mM, 20 μL per well) was pipetted onto a vacuum-centrifuge-dried 384-well plate with nanofractionated toxins using a Multidrop 384 Reagent Dispenser (Thermo Fisher Scientific, Ermelo, The Netherlands). After 3 min, plasma (20 μL per well) was also pipetted into the wells. The plate was then measured with a Varioskan^TM^ Flash Multimode Reader (Thermo Fisher Scientific, Ermelo, The Netherlands) for kinetic absorbance measurement at 595 nm at 25 °C over 80 min. The resulting coagulation activity data measured in each well were processed into two coagulation activities, which were procoagulation (0–15 min slope) and anticoagulation (80 min endpoint). Procoagulant or anticoagulant responses were plotted on the y-axis versus the retention time of fractionation on the *x*-axis, resulting in two coagulation chromatograms. Positive peaks in the procoagulant chromatograms resulted from procoagulant toxins, and negative peaks in the anticoagulation chromatograms represented anticoagulant toxins.

## Figures and Tables

**Table 1:** Comparison of the toxins found by LC-ESI-MS and LC-MALDI-MS. In the table, the following columns are given: the venom species, RT (retention time), *m*/*z* (mass-to-charge) value of the most intense charge state, the charge of the most intense charge state, the accurate mass only found in LC-ESI-MS (Da), the accurate mass intensity only found in LC-ESI-MS, the accurate mass (Da) found in LC-MALDI-MS that matched with the mass found in LC-ESI-MS, the mass intensity of MALDI-MS that was also found in the LC-ESI-MS data (i.e., the S/N signal extracted from the Bruker software 3.4), the mass difference between LC-ESI-MS and LC-MALDI-MS, the mass only found in LC-MALDI-MS, the intensity (i.e., the S/N signal extracted from the Bruker software 3.4) of the mass found in LC-MALDI-MS, and the percentage of the number of overlapped masses in both LC-ESI-MS and LC-MALDI-MS.

Venom Species	RT	*m*/*z*	Charge	Mass in ESI (Da)	Intensity	Mass in MALDI (Matched)	Intensity (*S*/*N*)	Mass diff (MALDI-ESI) (Da)	Mass in MALDI (Unmatched)	Intensity (*S*/*N*)	Mass Matching Coverage (%)
*Bitus arietans*	5.2–11.3	1400.2241	5	6992.07	12,306	7001	655	9	6570	237	25.00%
Figure 2	7.1–9.0	1396.6335	5	6976.09	1666	6966	607	10	8528	24	
	7.3–11.1	1795.9314	5	8983.59	107,914				7523	17	
	7.3–11.1	1802.9288	5	9003.58	14,870	9008	906	5	10,035	49	
	7.3–11.1	1807.943	5	9028.62	27,068				15,027	61	
	7.3–11.1	1762.1137	5	8799.51	16,208				6816	13	
	7.3–11.1	1807.943	5	9028.62	26,528				13,607	10	
	9.8–10.8	2374.5409	6	14,233.18	7828				30,072 (15,027^2+^)	21	
	9.8–10.8	2378.3701	6	14,257.15	1140				13,930 (6966^2+^)	222	
	12.5–13.7	1653.8255	8	13,213.51	1738	13,209	129	4	14,247 (7121^2+^)	22	
	12.5–13.7	2319.1642	6	13,901.89	8510						
	13.7–23.0	3334.0201	9	29,994.17	2114						
	13.7–23.0	1875.7795	12	22,495.54	1516						
	29.0–31.9	3326.3071	7	23,248.07	10,172						
	29.0–31.9	1663.7383	7	11,631.08	6924						
	31.8–36.7	3355.8697	7	23,476.06	2192						
*Bothrops jararaca*	7.0–10.0	1554.6483	5	7756.07	36,618	7766	929	10	11,657	8	27.50%
Figure 3	7.0–10.0	1936.0262	4	7735.06	3556				23,287	3	
	7.0–10.0	1464.6121	5	7313.01	8580	7309	277	4	7506	18	
	7.0–10.0	1504.6276	5	7514.07	4550	7505	18	9	7120	42	
	9.5–10.6	2766.5867	5	13,818.87	18,819			12	6851	55	
	9.5–10.6	2286.4299	6	13,704.51	19,268				6901	42	
	9.5–10.6	2758.799	5	13,780.39	5892				13,806 (6901^2+^)	14	
	10.5–14.7	2467.7336	10	24,658.28	4216				9463	3	
	14.8–21.2	2795.1658	9	25,142.31	3454				13,599	8	
	14.8–21.2	3315.5253	8	26,512.17	2066				9885	3	
	14.8–21.2	2036.9942	7	14,246.87	1794	14,240 (7119^2+^)	18	6.8	12,295	4	
	14.8–21.2	2281.9535	6	13,683.66	2650	13,684	38	0.44	16,367	6	
	20.9–24.0	2832.869	8	22,654.11	888				24,520	9	
	23.9–24.9	3298.4789	7	23,075.34	1402				48,925 (24,520^2+^)	8	
	24.9–26.2	3287.0082	7	22,995.04	3146						
	26.3–27.3	1037.0304	6	6211.12	11,672						
	26.3–27.3	1646.8429	5	8224.16	2262	8220	3	4.16			
	26.3–27.3	1769.5559	11	19,447.17	3096						
	26.3–27.3	2300.3188	10	22,978.01	3888						
	27.1–32.3	2897.6498	8	23,165.12	3588						
	32.2–37.1	2255.4149	11	24,778.20	3276						
	32.6–37.6	988.1154	10	9869.11	3034						
*Bothrops asper*	7.0–8.1	1441.6065	5	7198.98	26,068	7196	731	2.98	6964	2154	57.1
Figure 4	7.0–8.1	1518.8354	5	7585.12	22,106	7582	561	3.12	12,415	1438	
	7.0–8.1	1373.5657	5	6860.75	936	6863	1454	2.25	14,426	5	
	9.1–11.3	2323.5981	6	13,926.51	189,332	13,931	2868	5.5	6206	48	
	9.1–11.3	1717.8151	8	13,727.49	23,910	13,732 (6964^2+^)	997	5.6	9293	19	
	9.1–11.3	3097.7836	9	27,854.79	1676	27,844	55	10	8275	166	
	15.5–16.2	2364.5032	6	14,171.94	40,948						
	16.6–17.5	2316.5788	6	13,885.40	54,236						
	16.6–17.5	2328.5891	6	13,956.45	36,854	13,956 (6977^2+^)	222	0.45			
	16.6–17.5	3026.8793	9	27,227.83	886						
	16.6–17.5	3441.6186	8	27,517.92	1010						
	20.1–21.7	3107.3883	8	24,837.02	15,140	24,828	149	9			
	20.1–21.7	1031.5355	6	6180.17	5500						
*Crotalus Atrox*	6.9–9.5	1530.0267	5	7640.07	27,004				6805	2425	27.2
Figure 5	7.2–9.5	1518.422	5	7582.05	24,424	7578	1215	4	8576	90	
	10.1–11.4	2297.4253	6	13,770.49	99,050				7329	103	
	10.1–11.4	2303.9181	6	13,790.44	6602	13,794	2146	4	6902	260	
	10.1–11.4	1669.6686	8	13,343.26	3608				7131	567	
	10.1–11.4	2055.9636	12	24,650.40	4116				14,217	214	
	10.1–11.4	2466.7524	10	24,650.40	3812	24,660	11	10	13,435	437	
	11.4–13.7	1493.0124	3	4473.01	46,196	4478	632	5	26,829 (13,435^2+^)	247	
	11.4–13.7	2355.1224	6	14,210.65	3588	14,217	236	7.6	27,128	364	
	13.9–16.1	3356.8584	8	26,835.81	13,386	26,829	247	6.8	5116	30	
	16.1–18.1	2266.8271	6	13,587.88	20,302	13,586 (6805^2+^)	1844	1.8	6080	51	
	16.1–18.1	3448.2985	8	27,567.35	3540				6525	9	
	18.1–21.2	2264.1591	6	13,584.87	120,860	13,594 (6805^2+^)	4771	10	7769	22	
									6174	18	
									8225	49	
									12,329	62	
									24,660 (12,329^2+^)	11	
									9308	20	
									12,804	23	
									9055	30	
									40,662	20	
									27,518	35	
*Daboia russelli*	7.0–7.8	1729.7721	4	6911.04	3814				6822	1200	40
Figure 6	7.0–7.8	1861.076	4	7435.26	6572				13,622	2256	
	7.0–7.8	1864.3462	4	7447.32	5876	7448	227	1	6937	311	
	7.8–8.7	1931.0257	5	9644.06	13,522				8237	114	
	12.7–14.3	2059.6193	6	12,341.64	7308	12,347	228	6	7523	24	
	12.7–14.3	1650.1766	4	6589.66	1042	6600	30	10	9679	18	
	14.3–15.4	2277.1182	6	13,648.61	1208	13,644 (6822^2+^)	3098	4.6	12,973	3	
	15.5–18.5	2266.8742	6	13,589.18	507,424				14,559	21	
	15.5–18.5	3400.0414	8	27,176.18	33,140				54,273	8	
	22.3–23.7	2275.0336	6	13,635.12	148,486	13,633	2504	2.1			
	23.8–26.7	3415.5358	8	27,300.12	22,884	27,292	5	8			
*Echis carinatus*	5.7–7.4	1387.3333	4	5542.29	120,170	5553	1274	10	6795	1528	38.4
Figure 7	5.7–7.4	1312.05	4	5239.14	21,678	5239	1853	0.1	6898	2799	
	5.7–7.4	1350.5661	4	5387.18	17,486	5385	1241	2.1	7041	930	
	9.1–12.4	1726.2992	8	13,793.29	157,468	13,798	2729	4.8	9044	32	
	9.1–12.4	2716.5568	5	13,568.73	136,396				27,145	35	
	9.1–12.4	1512.3648	9	13,593.18	6742	13,590	1190	3.1			
	9.1–12.4	3068.1819	9	27,594.45	1974						
	12.7–16.8	2048.1235	12	24,559.34	3664						
	16.8–19.3	3315.5447	9	29,812.82	1722						
	19.0–20.3	3489.1665	10	34,879.59	718						
	20.0–22.3	2793.681	11	30,707.31	7790						
	34.3–37.1	1150.5924	4	4594.33	47,050						
	34.3–37.1	3308.1507	7	23,136.91	20,660						
*Echis Ocelattus*	5.8–7.6	1399.3083	4	5589.19	39,788	5585	997	4.1	6558	22	30
Figure 8	5.8–7.6	1705.6812	3	5111.01	5854	5113	406	2	6949	2275	
	7.2–9.2	2463.9245	10	24,626.11	560				7604	152	
	9.1–10.9	2305.0528	6	138,15.24	235,156	138,19	30,745	3.8	8206	10	
	11.0–13.4	2311.8515	6	138,56.03	81,606				9207	27	
	19.7–21.8	2764.4015	11	30,378.24	1656				10,434	37	
									12,316	16	
									15,494	31	
									24,645	16	
									27,617	190	

**Table 2:** Mass difference acceptance when analyzed with either only MALDI-MS or both ESI-MS and MALDI-MS.

Year	Analyte	Research Aims	Analyzer	Mass Cutoff (Da)	Reference
2003	Lipids	Investigate the phospholipid (PL) composition of organic extracts of bull spermatozoa	MALDI-TOF	16	[48]
2015	*Walterinnesia aegyptia*	Mass analysis to compare different separation and purification procedures	MALDI-TOF/TOF	8	[17]
2007	*Atheris squamigera*	Mass fingerprints for peptides	MALDI-TOF/MS and ESI-MS/MS	11	[49]
2005	*B. insularis* and *B. jararaca* venoms	Identify peptide content of crude snake venom	MALDI-TOF	9	[50]
2007	*Mesobuthus tamulus*	Mass fingerprints for biotope-specific variation	MALDI-TOF ESI-TOF	8	[51]
2008	*Opisthacanthus cayaporum*	Comparative analysis of amino acid sequence and biological activity for venom toxins	MALDI-TOF/TOF and ESI-MS/MS	8	[52]
2010	Enteric bacteria	Protein profile patterns and potential biomarkers for species and sub-species identification	MALDI-TOF/ ESI-TOF	10	[53]

## Data Availability

The data of superimposed MALDI-MS spectra of all toxin fractions analyzed for each venom for plotted MALDI chromatograms and argatroban (anticoagulant compound) analyzed by nanofractionation analytics for delay time calculation were provided in Supplementary Materials.

## Supplementary Materials

The following supporting information can be downloaded at: https://www.mdpi.com/article/10.3390/toxins16080370/s1, Figure S1: Superimposed MALDI-MS spectra of all toxin fractions analyzed for each venom.pptx; Figure S2: Argatroban (anticoagulant compound) analyzed by nanofractionation analytics.pptx.

## References

[1] Kasturiratne A., Wickremasinghe A.R., De Silva N., Gunawardena N.K., Pathmeswaran A., Premaratna R., Savioli L., Lalloo D.G., De Silva H.J. (2008). The global burden of snakebite: A literature analysis and modelling based on regional estimates of envenoming and deaths. PLoS Med..

[2] Williams D.J., Faiz M.A., Abela-Ridder B., Ainsworth S., Bulfone T.C., Nickerson A.D., Habib A.G., Junghanss T., Fan H.W., Turner M. (2019). Strategy for a globally coordinated response to a priority neglected tropical disease: Snakebite envenoming. PLoS Negl. Trop. Dis..

[3] Gutiérrez J.M., Calvete J.J., Habib A.G., Harrison R.A., Williams D.J., Warrell D.A. (2017). Snakebite envenoming. Nat. Rev. Dis. Primers.

[4] Lancet T. (2019). Snakebite—Emerging from the shadows of neglect. Lancet.

[5] Slagboom J., Kool J., Harrison R.A., Casewell N.R. (2017). Haemotoxic snake venoms: Their functional activity, impact on snakebite victims and pharmaceutical promise. Br. J. Haematol..

[6] Waheed H., Moin S.F., Choudhary M.I. (2017). Snake Venom: From Deadly Toxins to Life-saving Therapeutics. Curr. Med. Chem..

[7] Sanhajariya S., Duffull S.B., Isbister G.K. (2018). Pharmacokinetics of Snake Venom. Toxins.

[8] Tasoulis T., Isbister G.K. (2017). A Review and Database of Snake Venom Proteomes. Toxins.

[9] Kini R.M. (2011). Toxins in thrombosis and haemostasis: Potential beyond imagination. J. Thromb. Haemost..

[10] Kini R.M. (2006). Serine proteases affecting blood coagulation and fibrinolysis from snake venoms. Pathophysiol. Haemost. Thromb..

[11] Gutiérrez J.M., Escalante T., Rucavado A., Herrera C. (2016). Hemorrhage Caused by Snake Venom Metalloproteinases: A Journey of Discovery and Understanding. Toxins.

[12] Preciado L.M., Pereañez J.A., Comer J. (2019). Potential of Matrix Metalloproteinase Inhibitors for the Treatment of Local Tissue Damage Induced by a Type P-I Snake Venom Metalloproteinase. Toxins.

[13] Gutiérrez J.M., Rucavado A. (2000). Snake venom metalloproteinases: Their role in the pathogenesis of local tissue damage. Biochimie.

[14] Sunagar K., Jackson T.N.W., Reeks T., Fry B.G. (2015). Group I phospholipase A2 enzymes. Venomous Reptiles and Their Toxins: Evolution, Pathophysiology and Biodiscovery.

[15] Xiao H., Pan H., Liao K., Yang M., Huang C. (2017). Snake Venom PLA2, a Promising Target for Broad-Spectrum Antivenom Drug Development. BioMed Res. Int..

[16] Slagboom J., Kaal C., Arrahman A., Vonk F.J., Somsen G.W., Calvete J.J., Wüster W., Kool J. (2022). Analytical strategies in venomics. Microchem. J..

[17] El Aziz T.M.A., Bourgoin-Voillard S., Combemale S., Beroud R., Fadl M., Seve M., De Waard M. (2015). Fractionation and proteomic analysis of the Walterinnesia aegyptia snake venom using OFFGEL and MALDI-TOF-MS techniques. Electrophoresis.

[18] Brinkman D., Burnell J. (2008). Partial purification of cytolytic venom proteins from the box jellyfish, *Chironex fleckeri*. Toxicon.

[19] Carbajal-Saucedo A., López-Vera E., Bénard-Valle M., Smith E.N., Zamudio F., de Roodt A.R., Olvera-Rodríguez A. (2013). Isolation, characterization, cloning and expression of an alpha-neurotoxin from the venom of the Mexican coral snake Micrurus laticollaris (Squamata: Elapidae). Toxicon.

[20] Sánchez E.E., Soliz L.A., Ramírez M.S., Pérez J.C. (2001). Partial characterization of a basic protein from Crotalus molossus molossus (northern blacktail rattlesnake) venom and production of a monoclonal antibody. Toxicon.

[21] Slagboom J., Mladić M., Xie C., Kazandjian T.D., Vonk F., Somsen G.W., Casewell N.R., Kool J. (2020). High throughput screening and identification of coagulopathic snake venom proteins and peptides using nanofractionation and proteomics approaches. PLoS Negl. Trop. Dis..

[22] Xie C., Albulescu L.-O., Still K.B.M., Slagboom J., Zhao Y., Jiang Z., Somsen G.W., Vonk F.J., Casewell N.R., Kool J. (2020). Varespladib inhibits the phospholipase A2 and coagulopathic activities of venom components from hemotoxic snakes. Biomedicines.

[23] Mladic M., de Waal T., Burggraaff L., Slagboom J., Somsen G.W., Niessen W.M.A., Kini R.M., Kool J. (2017). Rapid screening and identification of ACE inhibitors in snake venoms using at-line nanofractionation LC-MS. Anal. Bioanal. Chem..

[24] Xie C., Bittenbinder M.A., Slagboom J., Arrahman A., Bruijns S., Somsen G.W., Vonk F.J., Casewell N.R., García-Vallejo J.J., Kool J. (2021). Erythrocyte haemotoxicity profiling of snake venom toxins after nanofractionation. J. Chromatogr. B Anal. Technol. Biomed. Life Sci..

[25] Bittenbinder M.A., Capinha L., Pereira D.D.C., Slagboom J., van de Velde B., Casewell N.R., Jennings P., Kool J., Vonk F.J. (2023). Development of a high-throughput in vitro screening method for the assessment of cell-damaging activities of snake venoms. PLoS Negl. Trop. Dis..

[26] Bickler P.E. (2020). Amplification of Snake Venom Toxicity by Endogenous Signaling Pathways. Toxins.

[27] Bohlen C.J., Chesler A.T., Sharif-Naeini R., Medzihradszky K.F., Zhou S., King D., Sánchez E.E., Burlingame A.L., Basbaum A.I., Julius D. (2011). A heteromeric Texas coral snake toxin targets acid-sensing ion channels to produce pain. Nature.

[28] Suntravat M., Sanchez O., Reyes A., Cirilo A., Ocheltree J.S., Galan J.A., Salazar E., Davies P., Sanchez E.E. (2021). Evaluation of signaling pathways profiling in human dermal endothelial cells treated by snake venom cysteine-rich secretory proteins (Svcrisps) from north american snakes using reverse phase protein array (rppa). Toxins.

[29] Gutiérrez J.M., Lomonte B., León G., Alape-Girón A., Flores-Díaz M., Sanz L., Angulo Y., Calvete J.J. (2009). Snake venomics and antivenomics: Proteomic tools in the design and control of antivenoms for the treatment of snakebite envenoming. J. Proteom..

[30] Fry B.G., Wüster W., Ramjan S.F.R., Jackson T., Martelli P., Kini R.M. (2003). Analysis of Colubroidea snake venoms by liquid chromatography with mass spectrometry: Evolutionary and toxinological implications. Rapid Commun. Mass Spectrom..

[31] Petras D., Heiss P., Süssmuth R.D., Calvete J.J. (2015). Venom proteomics of indonesian king cobra, ophiophagus hannah: Integrating top-down and bottom-up approaches. J. Proteome Res..

[32] Valente R.H., Nicolau C.A., Perales J., Neves-Ferreira A.G. (2016). Snake Venom Proteopeptidomics: What Lies Behind the Curtain. Venom Genomics and Proteomics.

[33] Wilm M. (2011). Principles of electrospray ionization. Mol. Cell. Proteom..

[34] Xie C., Albulescu L.-O., Bittenbinder M.A., Somsen G.W., Vonk F.J., Casewell N.R., Kool J. (2020). Neutralizing effects of small molecule inhibitors and metal chelators on coagulopathic Viperinae snake venom toxins. Biomedicines.

[35] Laustsen A.H. (2018). Guiding recombinant antivenom development by omics technologies. New Biotechnol..

[36] Vonk F.J., Casewell N.R., Henkel C.V., Heimberg A.M., Jansen H.J., McCleary R.J.R., Kerkkamp H.M.E., Vos R.A., Guerreiro I., Calvete J.J. (2013). The king cobra genome reveals dynamic gene evolution and adaptation in the snake venom system. Proc. Natl. Acad. Sci. USA.

[37] McCleary R.J., Kini R.M. (2013). Non-enzymatic proteins from snake venoms: A gold mine of pharmacological tools and drug leads. Toxicon.

[38] Boldrini-França J., Pinheiro-Junior E.L., Peigneur S., Pucca M.B., Cerni F.A., Borges R.J., Costa T.R., Carone S.E.I., Fontes M.R.d.M., Sampaio S.V. (2020). Beyond hemostasis: A snake venom serine protease with potassium channel blocking and potential antitumor activities. Sci. Rep..

[39] Wagstaff S.C., Sanz L., Juárez P., Harrison R.A., Calvete J.J. (2009). Combined snake venomics and venom gland transcriptomic analysis of the ocellated carpet viper, Echis ocellatus. J. Proteom..

[40] Alape-Girón A., Flores-Díaz M., Sanz L., Madrigal M., Escolano J., Sasa M., Calvete J.J. (2009). Studies on the venom proteome of Bothrops asper: Perspectives and applications. Toxicon.

[41] Calvete J.J., Fasoli E., Sanz L., Boschetti E., Righetti P.G. (2009). Exploring the venom proteome of the western diamondback rattlesnake, Crotalus atrox, via snake venomics and combinatorial peptide ligand library approaches. J. Proteome Res..

[42] Sharma M., Das D., Iyer J.K., Kini R.M., Doley R. (2015). Unveiling the complexities of Daboia russelii venom, a medically important snake of India, by tandem mass spectrometry. Toxicon.

[43] Dingwoke E.J., Adamude F.A., Mohamed G., Klein A., Salihu A., Abubakar M.S., Sallau A.B. (2021). Venom proteomic analysis of medically important Nigerian viper Echis ocellatus and Bitis arietans snake species. Biochem. Biophys. Rep..

[44] Zelanis A., Menezes M.C., Kitano E.S., Liberato T., Tashima A.K., Pinto A.F., Sherman N.E., Ho P.L., Fox J.W., Serrano S.M. (2016). Proteomic identification of gender molecular markers in Bothrops jararaca venom. J. Proteom..

[45] Patra A., Kalita B., Chanda A., Mukherjee A.K. (2017). Proteomics and antivenomics of Echis carinatus carinatus venom: Correlation with pharmacological properties and pathophysiology of envenomation. Sci. Rep..

[46] Damm M., Hempel B.-F., Süssmuth R.D. (2021). Old world vipers-a review about snake venom proteomics of viperinae and their variations. Toxins.

[47] Sajevic T., Leonardi A., Križaj I. (2011). Haemostatically active proteins in snake venoms. Toxicon.

[48] Schiller J., Müller K., Süß R., Arnhold J., Gey C., Herrmann A., Leßig J., Arnold K., Müller P. (2003). Analysis of the lipid composition of bull spermatozoa by MALDI-TOF mass spectrometry—A cautionary note. Phys. Lipids.

[49] Favreau P., Cheneval O., Menin L., Michalet S., Gaertner H., Principaud F., Thai R., Ménez A., Bulet P., Stöcklin R. (2007). The venom of the snake genus Atheris contains a new class of peptides with clusters of histidine and glycine residues. Rapid Commun. Mass Spectrom..

[50] Wermelinger L.S., Dutra D.L.S., Oliveira-Carvalho A.L., Soares M.R., Bloch C., Zingali R.B. (2005). Fast analysis of low molecular mass compounds present in snake venom: Identification of ten new pyroglutamate-containing peptides. Rapid Commun. Mass Spectrom..

[51] Newton K.A., Clench M.R., Deshmukh R., Jeyaseelan K., Strong P.N. (2007). Mass fingerprinting of toxic fractions from the venom of the Indian red scorpion, Mesobuthus tamulus: Biotope-specific variation in the expression of venom peptides. Rapid Commun. Mass Spectrom..

[52] Schwartz E.F., Camargos T.S., Zamudio F.Z., Silva L.P., Bloch C., Caixeta F., Schwartz C.A., Possani L.D. (2008). Mass spectrometry analysis, amino acid sequence and biological activity of venom components from the Brazilian scorpion Opisthacanthus cayaporum. Toxicon.

[53] Mott T.M., Everley R.A., Wyatt S.A., Toney D.M., Croley T.R. (2010). Comparison of MALDI-TOF/MS and LC-QTOF/MS methods for the identification of enteric bacteria. Int. J. Mass Spectrom..

