# Supplementary material for: Nanofractionation Analytics for Comparing MALDI-MS and ESI-MS Data of Viperidae Snake Venom Toxins
Source: Toxins (Basel). 2024 Aug 21;16(8):370. doi: 10.3390/toxins16080370 (PMC11360109; doi:10.3390/toxins16080370)
Supplement: Supplementary file 1 [file toxins-16-00370-s001.zip › Figure S1. Superimposed MALDI-MS spectra of all toxin fractions analyzed for each venom.pptx]

## Slide 1
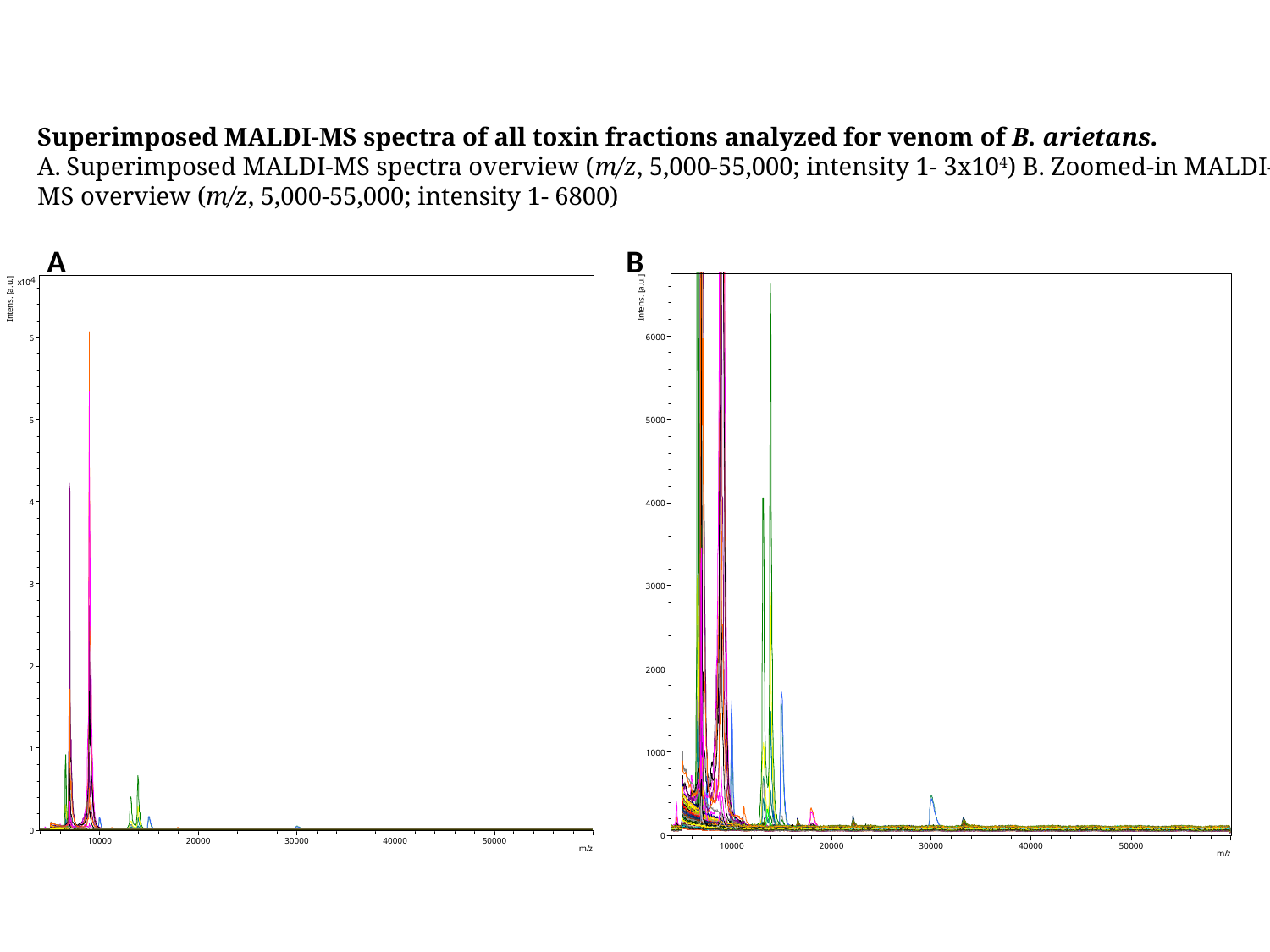

Superimposed MALDI-MS spectra of all toxin fractions analyzed for venom of B. arietans.
A. Superimposed MALDI-MS spectra overview (m/z, 5,000-55,000; intensity 1- 3x10^4^) B. Zoomed-in MALDI-MS overview (m/z, 5,000-55,000; intensity 1- 6800)
A
B

## Slide 2
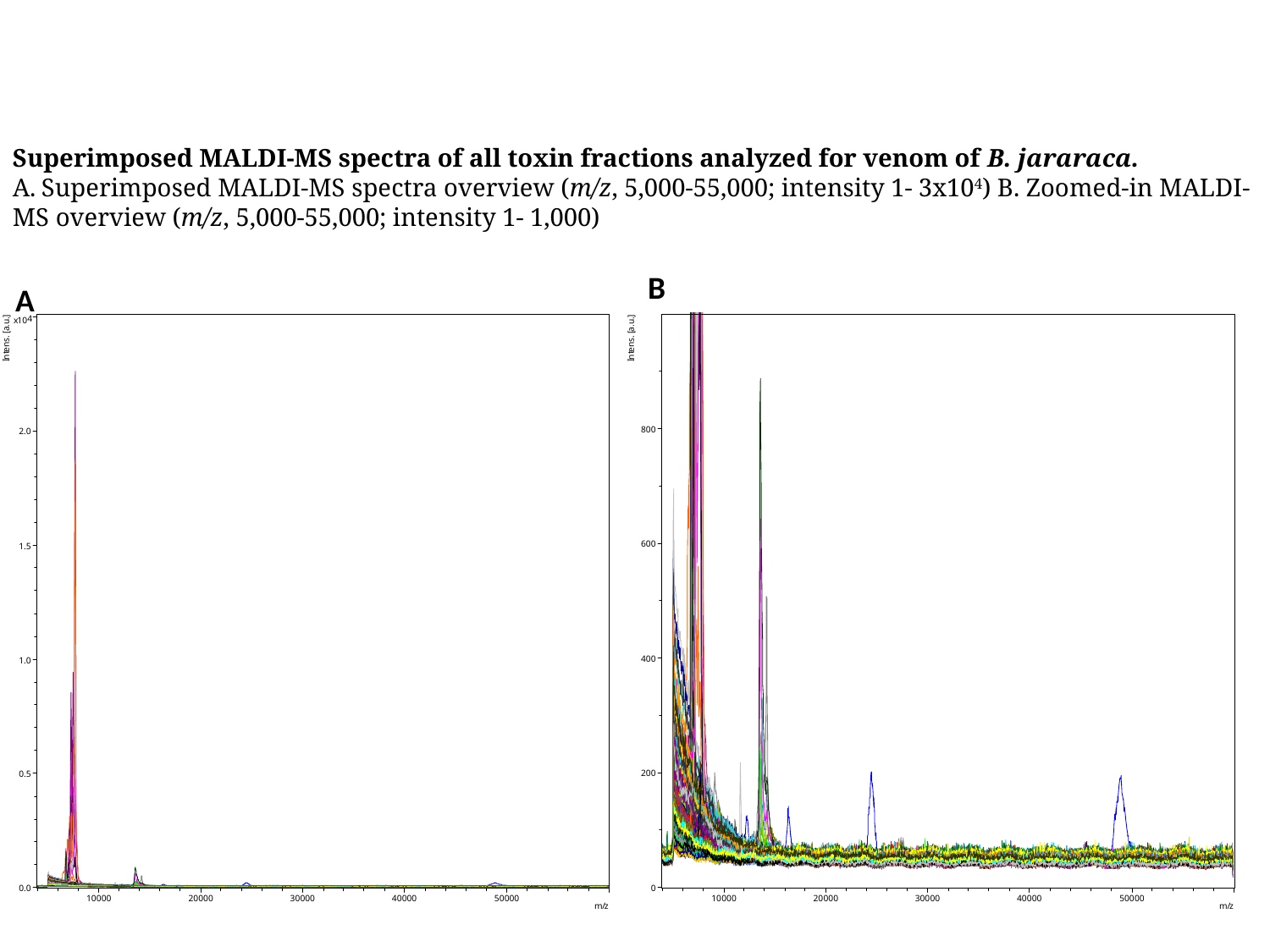

Superimposed MALDI-MS spectra of all toxin fractions analyzed for venom of B. jararaca.
A. Superimposed MALDI-MS spectra overview (m/z, 5,000-55,000; intensity 1- 3x10^4^) B. Zoomed-in MALDI-MS overview (m/z, 5,000-55,000; intensity 1- 1,000)
B
A

## Slide 3
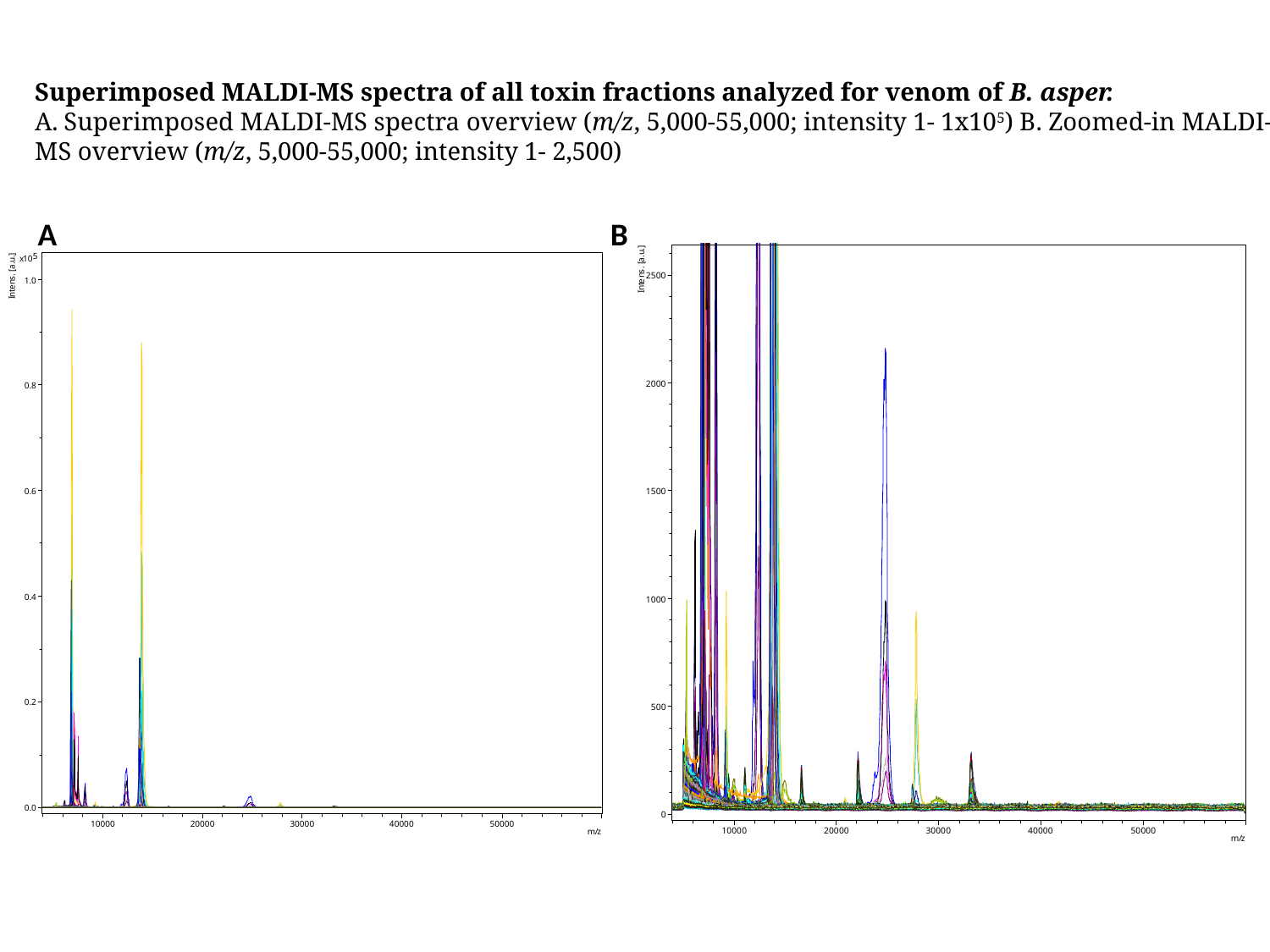

Superimposed MALDI-MS spectra of all toxin fractions analyzed for venom of B. asper.
A. Superimposed MALDI-MS spectra overview (m/z, 5,000-55,000; intensity 1- 1x10^5^) B. Zoomed-in MALDI-MS overview (m/z, 5,000-55,000; intensity 1- 2,500)
A
B

## Slide 4
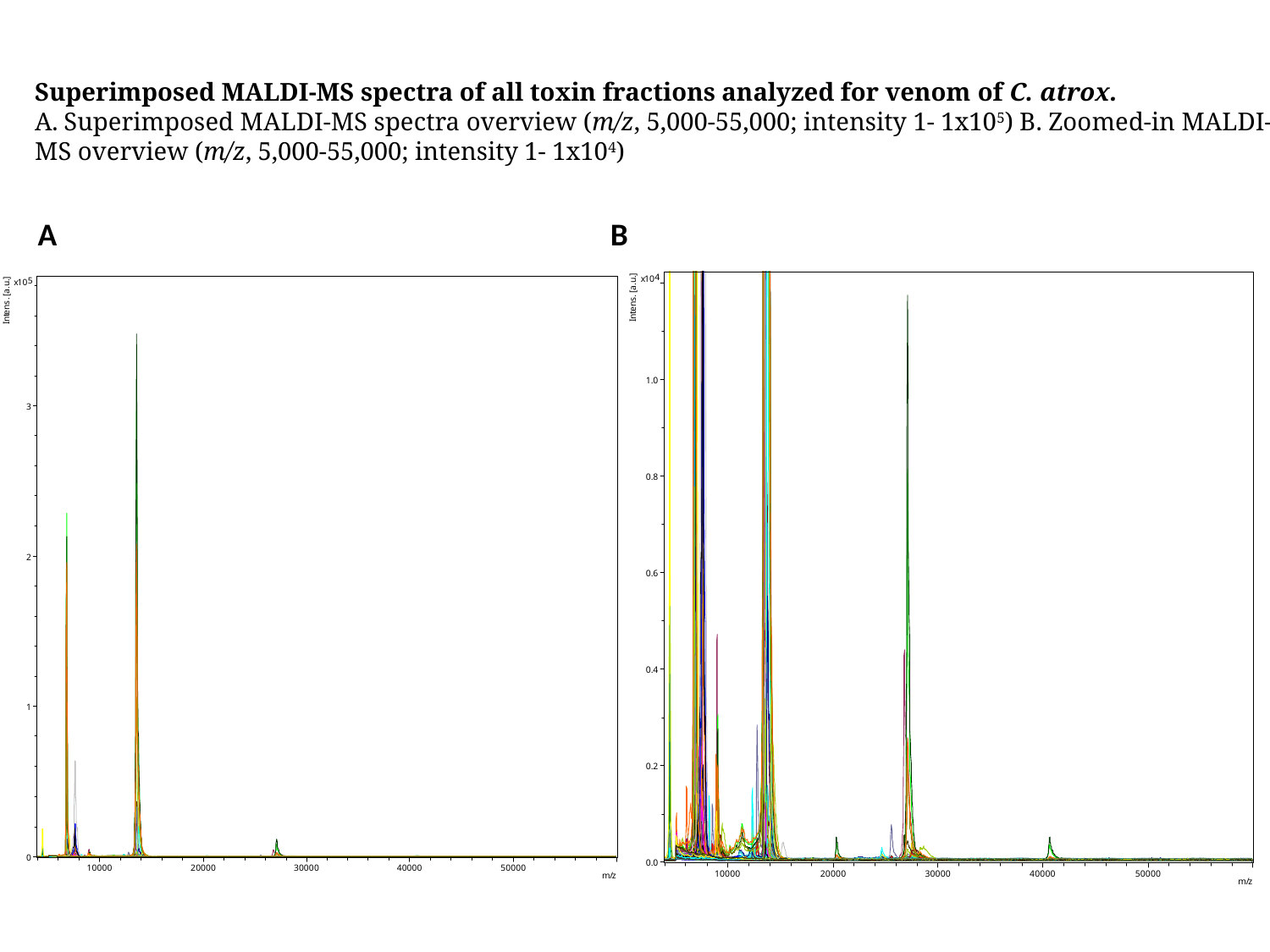

Superimposed MALDI-MS spectra of all toxin fractions analyzed for venom of C. atrox.
A. Superimposed MALDI-MS spectra overview (m/z, 5,000-55,000; intensity 1- 1x10^5^) B. Zoomed-in MALDI-MS overview (m/z, 5,000-55,000; intensity 1- 1x10^4^)
A
B

## Slide 5
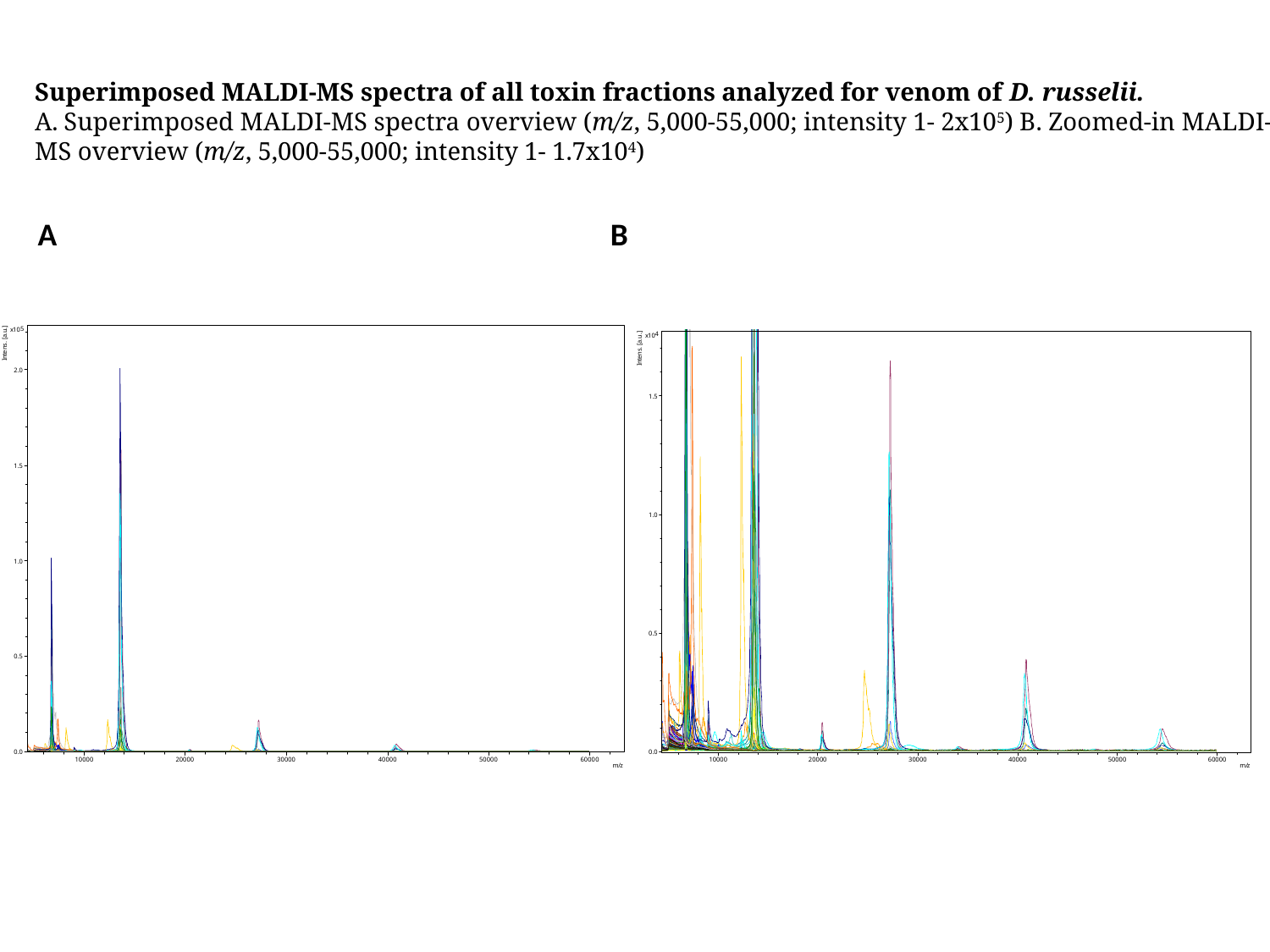

Superimposed MALDI-MS spectra of all toxin fractions analyzed for venom of D. russelii.
A. Superimposed MALDI-MS spectra overview (m/z, 5,000-55,000; intensity 1- 2x10^5^) B. Zoomed-in MALDI-MS overview (m/z, 5,000-55,000; intensity 1- 1.7x10^4^)
A
B

## Slide 6
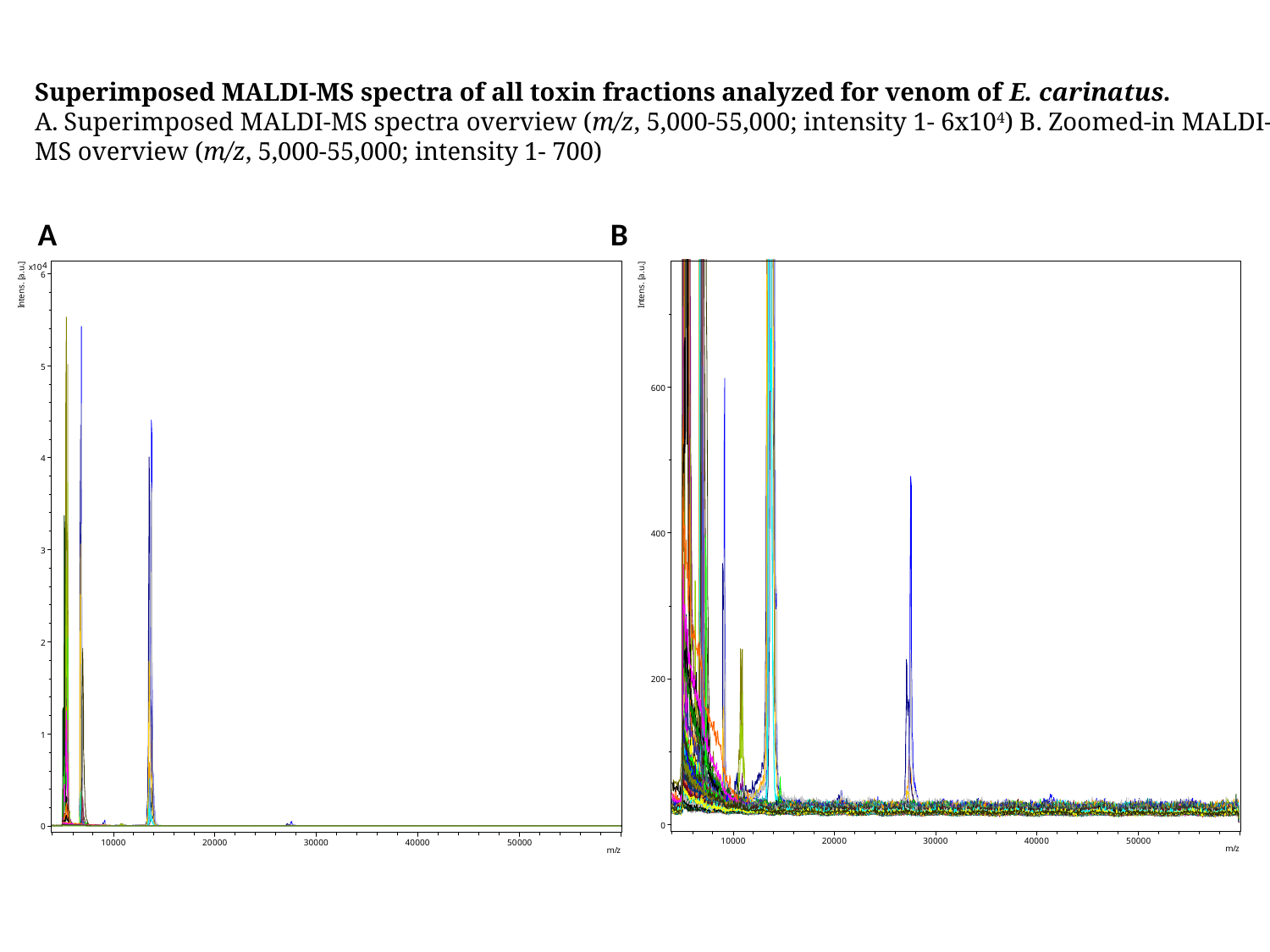

Superimposed MALDI-MS spectra of all toxin fractions analyzed for venom of E. carinatus.
A. Superimposed MALDI-MS spectra overview (m/z, 5,000-55,000; intensity 1- 6x10^4^) B. Zoomed-in MALDI-MS overview (m/z, 5,000-55,000; intensity 1- 700)
A
B

## Slide 7
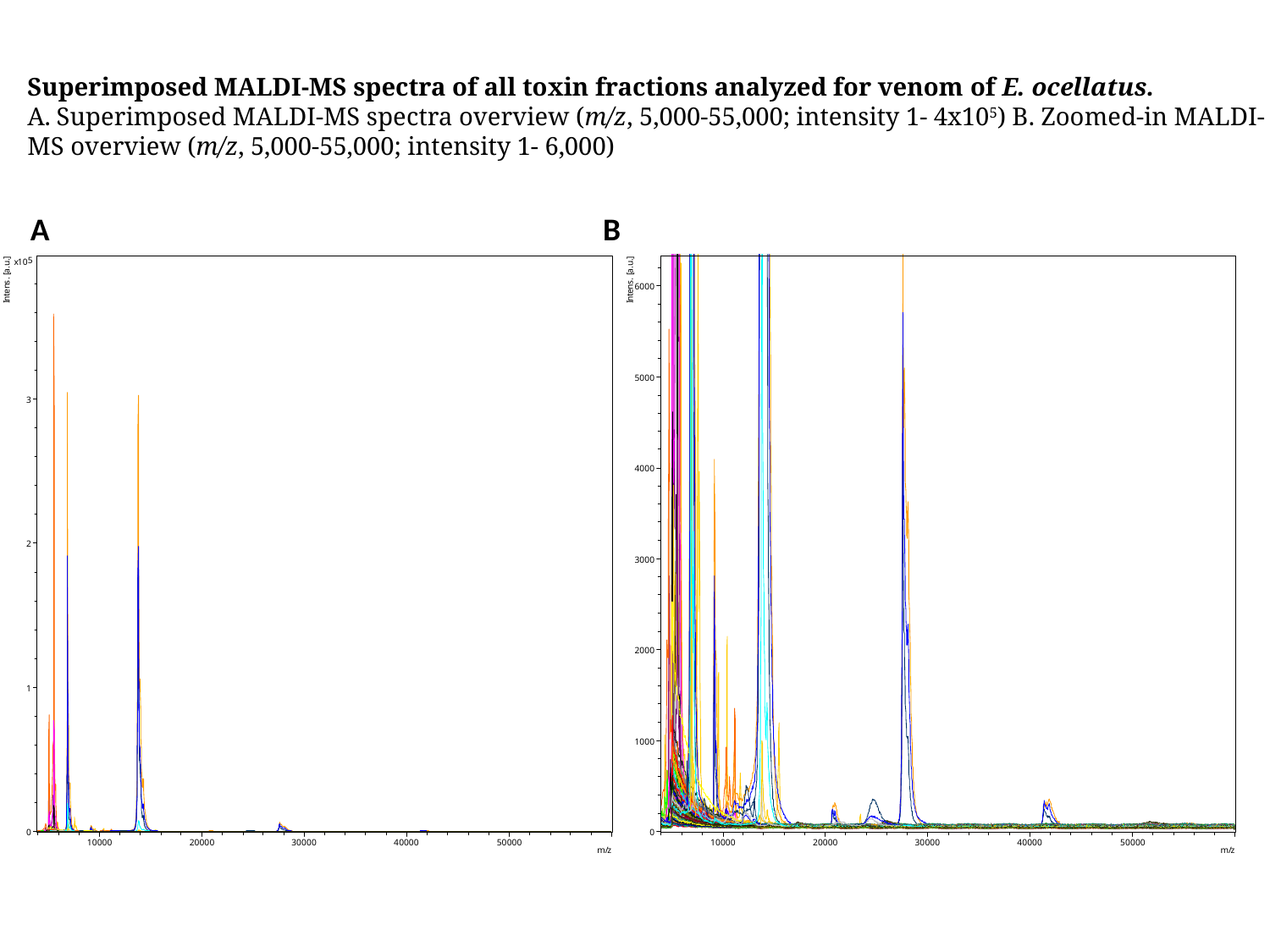

Superimposed MALDI-MS spectra of all toxin fractions analyzed for venom of E. ocellatus.
A. Superimposed MALDI-MS spectra overview (m/z, 5,000-55,000; intensity 1- 4x10^5^) B. Zoomed-in MALDI-MS overview (m/z, 5,000-55,000; intensity 1- 6,000)
A
B
